# Measurements of jet vetoes and azimuthal decorrelations in dijet events produced in $pp$ collisions at $\sqrt{s}=7\,\mathrm{TeV}$ using the ATLAS detector

**DOI:** 10.1140/epjc/s10052-014-3117-7

**Published:** 2014-10-31

**Authors:** The ATLAS Collaboration, G. Aad, B. Abbott, J. Abdallah, S. Abdel Khalek, O. Abdinov, R. Aben, B. Abi, M. Abolins, O. S. AbouZeid, H. Abramowicz, H. Abreu, R. Abreu, Y. Abulaiti, B. S. Acharya, L. Adamczyk, D. L. Adams, J. Adelman, S. Adomeit, T. Adye, T. Agatonovic-Jovin, J. A. Aguilar-Saavedra, M. Agustoni, S. P. Ahlen, F. Ahmadov, G. Aielli, H. Akerstedt, T. P. A. Åkesson, G. Akimoto, A. V. Akimov, G. L. Alberghi, J. Albert, S. Albrand, M. J. Alconada Verzini, M. Aleksa, I. N. Aleksandrov, C. Alexa, G. Alexander, G. Alexandre, T. Alexopoulos, M. Alhroob, G. Alimonti, L. Alio, J. Alison, B. M. M. Allbrooke, L. J. Allison, P. P. Allport, J. Almond, A. Aloisio, A. Alonso, F. Alonso, C. Alpigiani, A. Altheimer, B. Alvarez Gonzalez, M. G. Alviggi, K. Amako, Y. Amaral Coutinho, C. Amelung, D. Amidei, S. P. Amor Dos Santos, A. Amorim, S. Amoroso, N. Amram, G. Amundsen, C. Anastopoulos, L. S. Ancu, N. Andari, T. Andeen, C. F. Anders, G. Anders, K. J. Anderson, A. Andreazza, V. Andrei, X. S. Anduaga, S. Angelidakis, I. Angelozzi, P. Anger, A. Angerami, F. Anghinolfi, A. V. Anisenkov, N. Anjos, A. Annovi, A. Antonaki, M. Antonelli, A. Antonov, J. Antos, F. Anulli, M. Aoki, L. Aperio Bella, R. Apolle, G. Arabidze, I. Aracena, Y. Arai, J. P. Araque, A. T. H. Arce, J-F. Arguin, S. Argyropoulos, M. Arik, A. J. Armbruster, O. Arnaez, V. Arnal, H. Arnold, M. Arratia, O. Arslan, A. Artamonov, G. Artoni, S. Asai, N. Asbah, A. Ashkenazi, B. Åsman, L. Asquith, K. Assamagan, R. Astalos, M. Atkinson, N. B. Atlay, B. Auerbach, K. Augsten, M. Aurousseau, G. Avolio, G. Azuelos, Y. Azuma, M. A. Baak, A. Baas, C. Bacci, H. Bachacou, K. Bachas, M. Backes, M. Backhaus, J. Backus Mayes, E. Badescu, P. Bagiacchi, P. Bagnaia, Y. Bai, T. Bain, J. T. Baines, O. K. Baker, P. Balek, F. Balli, E. Banas, Sw. Banerjee, A. A. E. Bannoura, V. Bansal, H. S. Bansil, L. Barak, S. P. Baranov, E. L. Barberio, D. Barberis, M. Barbero, T. Barillari, M. Barisonzi, T. Barklow, N. Barlow, B. M. Barnett, R. M. Barnett, Z. Barnovska, A. Baroncelli, G. Barone, A. J. Barr, F. Barreiro, J. Barreiro Guimarães da Costa, R. Bartoldus, A. E. Barton, P. Bartos, V. Bartsch, A. Bassalat, A. Basye, R. L. Bates, J. R. Batley, M. Battaglia, M. Battistin, F. Bauer, H. S. Bawa, M. D. Beattie, T. Beau, P. H. Beauchemin, R. Beccherle, P. Bechtle, H. P. Beck, K. Becker, S. Becker, M. Beckingham, C. Becot, A. J. Beddall, A. Beddall, S. Bedikian, V. A. Bednyakov, C. P. Bee, L. J. Beemster, T. A. Beermann, M. Begel, K. Behr, C. Belanger-Champagne, P. J. Bell, W. H. Bell, G. Bella, L. Bellagamba, A. Bellerive, M. Bellomo, K. Belotskiy, O. Beltramello, O. Benary, D. Benchekroun, K. Bendtz, N. Benekos, Y. Benhammou, E. Benhar Noccioli, J. A. Benitez Garcia, D. P. Benjamin, J. R. Bensinger, K. Benslama, S. Bentvelsen, D. Berge, E. Bergeaas Kuutmann, N. Berger, F. Berghaus, J. Beringer, C. Bernard, P. Bernat, C. Bernius, F. U. Bernlochner, T. Berry, P. Berta, C. Bertella, G. Bertoli, F. Bertolucci, C. Bertsche, D. Bertsche, M. F. Bessner, M. I. Besana, G. J. Besjes, O. Bessidskaia, N. Besson, C. Betancourt, S. Bethke, W. Bhimji, R. M. Bianchi, L. Bianchini, M. Bianco, O. Biebel, S. P. Bieniek, K. Bierwagen, J. Biesiada, M. Biglietti, J. Bilbao De Mendizabal, H. Bilokon, M. Bindi, S. Binet, A. Bingul, C. Bini, C. W. Black, J. E. Black, K. M. Black, D. Blackburn, R. E. Blair, J.-B. Blanchard, T. Blazek, I. Bloch, C. Blocker, W. Blum, U. Blumenschein, G. J. Bobbink, V. S. Bobrovnikov, S. S. Bocchetta, A. Bocci, C. Bock, C. R. Boddy, M. Boehler, J. Boek, J. Boek, T. T. Boek, J. A. Bogaerts, A. G. Bogdanchikov, A. Bogouch, C. Bohm, J. Bohm, V. Boisvert, T. Bold, V. Boldea, A. S. Boldyrev, M. Bomben, M. Bona, M. Boonekamp, A. Borisov, G. Borissov, M. Borri, S. Borroni, J. Bortfeldt, V. Bortolotto, K. Bos, D. Boscherini, M. Bosman, H. Boterenbrood, J. Boudreau, J. Bouffard, E. V. Bouhova-Thacker, D. Boumediene, C. Bourdarios, N. Bousson, S. Boutouil, A. Boveia, J. Boyd, I. R. Boyko, J. Bracinik, A. Brandt, G. Brandt, O. Brandt, U. Bratzler, B. Brau, J. E. Brau, H. M. Braun, S. F. Brazzale, B. Brelier, K. Brendlinger, A. J. Brennan, R. Brenner, S. Bressler, K. Bristow, T. M. Bristow, D. Britton, F. M. Brochu, I. Brock, R. Brock, C. Bromberg, J. Bronner, G. Brooijmans, T. Brooks, W. K. Brooks, J. Brosamer, E. Brost, J. Brown, P. A. Bruckman de Renstrom, D. Bruncko, R. Bruneliere, S. Brunet, A. Bruni, G. Bruni, M. Bruschi, L. Bryngemark, T. Buanes, Q. Buat, F. Bucci, P. Buchholz, R. M. Buckingham, A. G. Buckley, S. I. Buda, I. A. Budagov, F. Buehrer, L. Bugge, M. K. Bugge, O. Bulekov, A. C. Bundock, H. Burckhart, S. Burdin, B. Burghgrave, S. Burke, I. Burmeister, E. Busato, D. Büscher, V. Büscher, P. Bussey, C. P. Buszello, B. Butler, J. M. Butler, A. I. Butt, C. M. Buttar, J. M. Butterworth, P. Butti, W. Buttinger, A. Buzatu, M. Byszewski, S. Cabrera Urbán, D. Caforio, O. Cakir, P. Calafiura, A. Calandri, G. Calderini, P. Calfayan, R. Calkins, L. P. Caloba, D. Calvet, S. Calvet, R. Camacho Toro, S. Camarda, D. Cameron, L. M. Caminada, R. Caminal Armadans, S. Campana, M. Campanelli, A. Campoverde, V. Canale, A. Canepa, M. Cano Bret, J. Cantero, R. Cantrill, T. Cao, M. D. M. Capeans Garrido, I. Caprini, M. Caprini, M. Capua, R. Caputo, R. Cardarelli, T. Carli, G. Carlino, L. Carminati, S. Caron, E. Carquin, G. D. Carrillo-Montoya, J. R. Carter, J. Carvalho, D. Casadei, M. P. Casado, M. Casolino, E. Castaneda-Miranda, A. Castelli, V. Castillo Gimenez, N. F. Castro, P. Catastini, A. Catinaccio, J. R. Catmore, A. Cattai, G. Cattani, S. Caughron, V. Cavaliere, D. Cavalli, M. Cavalli-Sforza, V. Cavasinni, F. Ceradini, B. Cerio, K. Cerny, A. S. Cerqueira, A. Cerri, L. Cerrito, F. Cerutti, M. Cerv, A. Cervelli, S. A. Cetin, A. Chafaq, D. Chakraborty, I. Chalupkova, P. Chang, B. Chapleau, J. D. Chapman, D. Charfeddine, D. G. Charlton, C. C. Chau, C. A. Chavez Barajas, S. Cheatham, A. Chegwidden, S. Chekanov, S. V. Chekulaev, G. A. Chelkov, M. A. Chelstowska, C. Chen, H. Chen, K. Chen, L. Chen, S. Chen, X. Chen, Y. Chen, Y. Chen, H. C. Cheng, Y. Cheng, A. Cheplakov, R. Cherkaoui El Moursli, V. Chernyatin, E. Cheu, L. Chevalier, V. Chiarella, G. Chiefari, J. T. Childers, A. Chilingarov, G. Chiodini, A. S. Chisholm, R. T. Chislett, A. Chitan, M. V. Chizhov, S. Chouridou, B. K. B. Chow, D. Chromek-Burckhart, M. L. Chu, J. Chudoba, J. J. Chwastowski, L. Chytka, G. Ciapetti, A. K. Ciftci, R. Ciftci, D. Cinca, V. Cindro, A. Ciocio, P. Cirkovic, Z. H. Citron, M. Citterio, M. Ciubancan, A. Clark, P. J. Clark, R. N. Clarke, W. Cleland, J. C. Clemens, C. Clement, Y. Coadou, M. Cobal, A. Coccaro, J. Cochran, L. Coffey, J. G. Cogan, J. Coggeshall, B. Cole, S. Cole, A. P. Colijn, J. Collot, T. Colombo, G. Colon, G. Compostella, P. Conde Muiño, E. Coniavitis, M. C. Conidi, S. H. Connell, I. A. Connelly, S. M. Consonni, V. Consorti, S. Constantinescu, C. Conta, G. Conti, F. Conventi, M. Cooke, B. D. Cooper, A. M. Cooper-Sarkar, N. J. Cooper-Smith, K. Copic, T. Cornelissen, M. Corradi, F. Corriveau, A. Corso-Radu, A. Cortes-Gonzalez, G. Cortiana, G. Costa, M. J. Costa, D. Costanzo, D. Côté, G. Cottin, G. Cowan, B. E. Cox, K. Cranmer, G. Cree, S. Crépé-Renaudin, F. Crescioli, W. A. Cribbs, M. Crispin Ortuzar, M. Cristinziani, V. Croft, G. Crosetti, C.-M. Cuciuc, T. Cuhadar Donszelmann, J. Cummings, M. Curatolo, C. Cuthbert, H. Czirr, P. Czodrowski, Z. Czyczula, S. D’Auria, M. D’Onofrio, M. J. Da Cunha Sargedas De Sousa, C. Da Via, W. Dabrowski, A. Dafinca, T. Dai, O. Dale, F. Dallaire, C. Dallapiccola, M. Dam, A. C. Daniells, M. Dano Hoffmann, V. Dao, G. Darbo, S. Darmora, J. A. Dassoulas, A. Dattagupta, W. Davey, C. David, T. Davidek, E. Davies, M. Davies, O. Davignon, A. R. Davison, P. Davison, Y. Davygora, E. Dawe, I. Dawson, R. K. Daya-Ishmukhametova, K. De, R. de Asmundis, S. De Castro, S. De Cecco, N. De Groot, P. de Jong, H. De la Torre, F. De Lorenzi, L. De Nooij, D. De Pedis, A. De Salvo, U. De Sanctis, A. De Santo, J. B. De Vivie De Regie, W. J. Dearnaley, R. Debbe, C. Debenedetti, B. Dechenaux, D. V. Dedovich, I. Deigaard, J. Del Peso, T. Del Prete, F. Deliot, C. M. Delitzsch, M. Deliyergiyev, A. Dell’Acqua, L. Dell’Asta, M. Dell’Orso, M. Della Pietra, D. della Volpe, M. Delmastro, P. A. Delsart, C. Deluca, S. Demers, M. Demichev, A. Demilly, S. P. Denisov, D. Derendarz, J. E. Derkaoui, F. Derue, P. Dervan, K. Desch, C. Deterre, P. O. Deviveiros, A. Dewhurst, S. Dhaliwal, A. Di Ciaccio, L. Di Ciaccio, A. Di Domenico, C. Di Donato, A. Di Girolamo, B. Di Girolamo, A. Di Mattia, B. Di Micco, R. Di Nardo, A. Di Simone, R. Di Sipio, D. Di Valentino, F. A. Dias, M. A. Diaz, E. B. Diehl, J. Dietrich, T. A. Dietzsch, S. Diglio, A. Dimitrievska, J. Dingfelder, C. Dionisi, P. Dita, S. Dita, F. Dittus, F. Djama, T. Djobava, M. A. B. do Vale, A. Do Valle Wemans, T. K. O. Doan, D. Dobos, C. Doglioni, T. Doherty, T. Dohmae, J. Dolejsi, Z. Dolezal, B. A. Dolgoshein, M. Donadelli, S. Donati, P. Dondero, J. Donini, J. Dopke, A. Doria, M. T. Dova, A. T. Doyle, M. Dris, J. Dubbert, S. Dube, E. Dubreuil, E. Duchovni, G. Duckeck, O. A. Ducu, D. Duda, A. Dudarev, F. Dudziak, L. Duflot, L. Duguid, M. Dührssen, M. Dunford, H. Duran Yildiz, M. Düren, A. Durglishvili, M. Dwuznik, M. Dyndal, J. Ebke, W. Edson, N. C. Edwards, W. Ehrenfeld, T. Eifert, G. Eigen, K. Einsweiler, T. Ekelof, M. El Kacimi, M. Ellert, S. Elles, F. Ellinghaus, N. Ellis, J. Elmsheuser, M. Elsing, D. Emeliyanov, Y. Enari, O. C. Endner, M. Endo, R. Engelmann, J. Erdmann, A. Ereditato, D. Eriksson, G. Ernis, J. Ernst, M. Ernst, J. Ernwein, D. Errede, S. Errede, E. Ertel, M. Escalier, H. Esch, C. Escobar, B. Esposito, A. I. Etienvre, E. Etzion, H. Evans, A. Ezhilov, L. Fabbri, G. Facini, R. M. Fakhrutdinov, S. Falciano, R. J. Falla, J. Faltova, Y. Fang, M. Fanti, A. Farbin, A. Farilla, T. Farooque, S. Farrell, S. M. Farrington, P. Farthouat, F. Fassi, P. Fassnacht, D. Fassouliotis, A. Favareto, L. Fayard, P. Federic, O. L. Fedin, W. Fedorko, M. Fehling-Kaschek, S. Feigl, L. Feligioni, C. Feng, E. J. Feng, H. Feng, A. B. Fenyuk, S. Fernandez Perez, S. Ferrag, J. Ferrando, A. Ferrari, P. Ferrari, R. Ferrari, D. E. Ferreira de Lima, A. Ferrer, D. Ferrere, C. Ferretti, A. Ferretto Parodi, M. Fiascaris, F. Fiedler, A. Filipčič, M. Filipuzzi, F. Filthaut, M. Fincke-Keeler, K. D. Finelli, M. C. N. Fiolhais, L. Fiorini, A. Firan, A. Fischer, J. Fischer, W. C. Fisher, E. A. Fitzgerald, M. Flechl, I. Fleck, P. Fleischmann, S. Fleischmann, G. T. Fletcher, G. Fletcher, T. Flick, A. Floderus, L. R. Flores Castillo, A. C. Florez Bustos, M. J. Flowerdew, A. Formica, A. Forti, D. Fortin, D. Fournier, H. Fox, S. Fracchia, P. Francavilla, M. Franchini, S. Franchino, D. Francis, L. Franconi, M. Franklin, S. Franz, M. Fraternali, S. T. French, C. Friedrich, F. Friedrich, D. Froidevaux, J. A. Frost, C. Fukunaga, E. Fullana Torregrosa, B. G. Fulsom, J. Fuster, C. Gabaldon, O. Gabizon, A. Gabrielli, A. Gabrielli, S. Gadatsch, S. Gadomski, G. Gagliardi, P. Gagnon, C. Galea, B. Galhardo, E. J. Gallas, V. Gallo, B. J. Gallop, P. Gallus, G. Galster, K. K. Gan, J. Gao, Y. S. Gao, F. M. Garay Walls, F. Garberson, C. García, J. E. García Navarro, M. Garcia-Sciveres, R. W. Gardner, N. Garelli, V. Garonne, C. Gatti, G. Gaudio, B. Gaur, L. Gauthier, P. Gauzzi, I. L. Gavrilenko, C. Gay, G. Gaycken, E. N. Gazis, P. Ge, Z. Gecse, C. N. P. Gee, D. A. A. Geerts, Ch. Geich-Gimbel, K. Gellerstedt, C. Gemme, A. Gemmell, M. H. Genest, S. Gentile, M. George, S. George, D. Gerbaudo, A. Gershon, H. Ghazlane, N. Ghodbane, B. Giacobbe, S. Giagu, V. Giangiobbe, P. Giannetti, F. Gianotti, B. Gibbard, S. M. Gibson, M. Gilchriese, T. P. S. Gillam, D. Gillberg, G. Gilles, D. M. Gingrich, N. Giokaris, M. P. Giordani, R. Giordano, F. M. Giorgi, F. M. Giorgi, P. F. Giraud, D. Giugni, C. Giuliani, M. Giulini, B. K. Gjelsten, S. Gkaitatzis, I. Gkialas, L. K. Gladilin, C. Glasman, J. Glatzer, P. C. F. Glaysher, A. Glazov, G. L. Glonti, M. Goblirsch-Kolb, J. R. Goddard, J. Godfrey, J. Godlewski, C. Goeringer, S. Goldfarb, T. Golling, D. Golubkov, A. Gomes, L. S. Gomez Fajardo, R. Gonçalo, J. Goncalves Pinto Firmino Da Costa, L. Gonella, S. González de la Hoz, G. Gonzalez Parra, S. Gonzalez-Sevilla, L. Goossens, P. A. Gorbounov, H. A. Gordon, I. Gorelov, B. Gorini, E. Gorini, A. Gorišek, E. Gornicki, A. T. Goshaw, C. Gössling, M. I. Gostkin, M. Gouighri, D. Goujdami, M. P. Goulette, A. G. Goussiou, C. Goy, S. Gozpinar, H. M. X. Grabas, L. Graber, I. Grabowska-Bold, P. Grafström, K.-J. Grahn, J. Gramling, E. Gramstad, S. Grancagnolo, V. Grassi, V. Gratchev, H. M. Gray, E. Graziani, O. G. Grebenyuk, Z. D. Greenwood, K. Gregersen, I. M. Gregor, P. Grenier, J. Griffiths, A. A. Grillo, K. Grimm, S. Grinstein, Ph. Gris, Y. V. Grishkevich, J.-F. Grivaz, J. P. Grohs, A. Grohsjean, E. Gross, J. Grosse-Knetter, G. C. Grossi, J. Groth-Jensen, Z. J. Grout, L. Guan, F. Guescini, D. Guest, O. Gueta, C. Guicheney, E. Guido, T. Guillemin, S. Guindon, U. Gul, C. Gumpert, J. Gunther, J. Guo, S. Gupta, P. Gutierrez, N. G. Gutierrez Ortiz, C. Gutschow, N. Guttman, C. Guyot, C. Gwenlan, C. B. Gwilliam, A. Haas, C. Haber, H. K. Hadavand, N. Haddad, P. Haefner, S. Hageböeck, Z. Hajduk, H. Hakobyan, M. Haleem, D. Hall, G. Halladjian, K. Hamacher, P. Hamal, K. Hamano, M. Hamer, A. Hamilton, S. Hamilton, G. N. Hamity, P. G. Hamnett, L. Han, K. Hanagaki, K. Hanawa, M. Hance, P. Hanke, R. Hann, J. B. Hansen, J. D. Hansen, P. H. Hansen, K. Hara, A. S. Hard, T. Harenberg, F. Hariri, S. Harkusha, D. Harper, R. D. Harrington, O. M. Harris, P. F. Harrison, F. Hartjes, M. Hasegawa, S. Hasegawa, Y. Hasegawa, A. Hasib, S. Hassani, S. Haug, M. Hauschild, R. Hauser, M. Havranek, C. M. Hawkes, R. J. Hawkings, A. D. Hawkins, T. Hayashi, D. Hayden, C. P. Hays, H. S. Hayward, S. J. Haywood, S. J. Head, T. Heck, V. Hedberg, L. Heelan, S. Heim, T. Heim, B. Heinemann, L. Heinrich, J. Hejbal, L. Helary, C. Heller, M. Heller, S. Hellman, D. Hellmich, C. Helsens, J. Henderson, Y. Heng, R. C. W. Henderson, C. Hengler, A. Henrichs, A. M. Henriques Correia, S. Henrot-Versille, C. Hensel, G. H. Herbert, Y. Hernández Jiménez, R. Herrberg-Schubert, G. Herten, R. Hertenberger, L. Hervas, G. G. Hesketh, N. P. Hessey, R. Hickling, E. Higón-Rodriguez, E. Hill, J. C. Hill, K. H. Hiller, S. Hillert, S. J. Hillier, I. Hinchliffe, E. Hines, M. Hirose, D. Hirschbuehl, J. Hobbs, N. Hod, M. C. Hodgkinson, P. Hodgson, A. Hoecker, M. R. Hoeferkamp, F. Hoenig, J. Hoffman, D. Hoffmann, J. I. Hofmann, M. Hohlfeld, T. R. Holmes, T. M. Hong, L. Hooft van Huysduynen, Y. Horii, J.-Y. Hostachy, S. Hou, A. Hoummada, J. Howard, J. Howarth, M. Hrabovsky, I. Hristova, J. Hrivnac, T. Hryn’ova, C. Hsu, P. J. Hsu, S.-C. Hsu, D. Hu, X. Hu, Y. Huang, Z. Hubacek, F. Hubaut, F. Huegging, T. B. Huffman, E. W. Hughes, G. Hughes, M. Huhtinen, T. A. Hülsing, M. Hurwitz, N. Huseynov, J. Huston, J. Huth, G. Iacobucci, G. Iakovidis, I. Ibragimov, L. Iconomidou-Fayard, E. Ideal, P. Iengo, O. Igonkina, T. Iizawa, Y. Ikegami, K. Ikematsu, M. Ikeno, Y. Ilchenko, D. Iliadis, N. Ilic, Y. Inamaru, T. Ince, P. Ioannou, M. Iodice, K. Iordanidou, V. Ippolito, A. Irles Quiles, C. Isaksson, M. Ishino, M. Ishitsuka, R. Ishmukhametov, C. Issever, S. Istin, J. M. Iturbe Ponce, R. Iuppa, J. Ivarsson, W. Iwanski, H. Iwasaki, J. M. Izen, V. Izzo, B. Jackson, M. Jackson, P. Jackson, M. R. Jaekel, V. Jain, K. Jakobs, S. Jakobsen, T. Jakoubek, J. Jakubek, D. O. Jamin, D. K. Jana, E. Jansen, H. Jansen, J. Janssen, M. Janus, G. Jarlskog, N. Javadov, T. Javůrek, L. Jeanty, J. Jejelava, G.-Y. Jeng, D. Jennens, P. Jenni, J. Jentzsch, C. Jeske, S. Jézéquel, H. Ji, J. Jia, Y. Jiang, M. Jimenez Belenguer, S. Jin, A. Jinaru, O. Jinnouchi, M. D. Joergensen, K. E. Johansson, P. Johansson, K. A. Johns, K. Jon-And, G. Jones, R. W. L. Jones, T. J. Jones, J. Jongmanns, P. M. Jorge, K. D. Joshi, J. Jovicevic, X. Ju, C. A. Jung, R. M. Jungst, P. Jussel, A. Juste Rozas, M. Kaci, A. Kaczmarska, M. Kado, H. Kagan, M. Kagan, E. Kajomovitz, C. W. Kalderon, S. Kama, A. Kamenshchikov, N. Kanaya, M. Kaneda, S. Kaneti, V. A. Kantserov, J. Kanzaki, B. Kaplan, A. Kapliy, D. Kar, K. Karakostas, N. Karastathis, M. Karnevskiy, S. N. Karpov, Z. M. Karpova, K. Karthik, V. Kartvelishvili, A. N. Karyukhin, L. Kashif, G. Kasieczka, R. D. Kass, A. Kastanas, Y. Kataoka, A. Katre, J. Katzy, V. Kaushik, K. Kawagoe, T. Kawamoto, G. Kawamura, S. Kazama, V. F. Kazanin, M. Y. Kazarinov, R. Keeler, R. Kehoe, M. Keil, J. S. Keller, J. J. Kempster, H. Keoshkerian, O. Kepka, B. P. Kerševan, S. Kersten, K. Kessoku, J. Keung, F. Khalil-zada, H. Khandanyan, A. Khanov, A. Khodinov, A. Khomich, T. J. Khoo, G. Khoriauli, A. Khoroshilov, V. Khovanskiy, E. Khramov, J. Khubua, H. Y. Kim, H. Kim, S. H. Kim, N. Kimura, O. Kind, B. T. King, M. King, R. S. B. King, S. B. King, J. Kirk, A. E. Kiryunin, T. Kishimoto, D. Kisielewska, F. Kiss, T. Kittelmann, K. Kiuchi, E. Kladiva, M. Klein, U. Klein, K. Kleinknecht, P. Klimek, A. Klimentov, R. Klingenberg, J. A. Klinger, T. Klioutchnikova, P. F. Klok, E.-E. Kluge, P. Kluit, S. Kluth, E. Kneringer, E. B. F. G. Knoops, A. Knue, D. Kobayashi, T. Kobayashi, M. Kobel, M. Kocian, P. Kodys, P. Koevesarki, T. Koffas, E. Koffeman, L. A. Kogan, S. Kohlmann, Z. Kohout, T. Kohriki, T. Koi, H. Kolanoski, I. Koletsou, J. Koll, A. A. Komar, Y. Komori, T. Kondo, N. Kondrashova, K. Köneke, A. C. König, S. König, T. Kono, R. Konoplich, N. Konstantinidis, R. Kopeliansky, S. Koperny, L. Köpke, A. K. Kopp, K. Korcyl, K. Kordas, A. Korn, A. A. Korol, I. Korolkov, E. V. Korolkova, V. A. Korotkov, O. Kortner, S. Kortner, V. V. Kostyukhin, V. M. Kotov, A. Kotwal, C. Kourkoumelis, V. Kouskoura, A. Koutsman, R. Kowalewski, T. Z. Kowalski, W. Kozanecki, A. S. Kozhin, V. Kral, V. A. Kramarenko, G. Kramberger, D. Krasnopevtsev, M. W. Krasny, A. Krasznahorkay, J. K. Kraus, A. Kravchenko, S. Kreiss, M. Kretz, J. Kretzschmar, K. Kreutzfeldt, P. Krieger, K. Kroeninger, H. Kroha, J. Kroll, J. Kroseberg, J. Krstic, U. Kruchonak, H. Krüger, T. Kruker, N. Krumnack, Z. V. Krumshteyn, A. Kruse, M. C. Kruse, M. Kruskal, T. Kubota, S. Kuday, S. Kuehn, A. Kugel, A. Kuhl, T. Kuhl, V. Kukhtin, Y. Kulchitsky, S. Kuleshov, M. Kuna, J. Kunkle, A. Kupco, H. Kurashige, Y. A. Kurochkin, R. Kurumida, V. Kus, E. S. Kuwertz, M. Kuze, J. Kvita, A. La Rosa, L. La Rotonda, C. Lacasta, F. Lacava, J. Lacey, H. Lacker, D. Lacour, V. R. Lacuesta, E. Ladygin, R. Lafaye, B. Laforge, T. Lagouri, S. Lai, H. Laier, L. Lambourne, S. Lammers, C. L. Lampen, W. Lampl, E. Lançon, U. Landgraf, M. P. J. Landon, V. S. Lang, A. J. Lankford, F. Lanni, K. Lantzsch, S. Laplace, C. Lapoire, J. F. Laporte, T. Lari, M. Lassnig, P. Laurelli, W. Lavrijsen, A. T. Law, P. Laycock, O. Le Dortz, E. Le Guirriec, E. Le Menedeu, T. LeCompte, F. Ledroit-Guillon, C. A. Lee, H. Lee, J. S. H. Lee, S. C. Lee, L. Lee, G. Lefebvre, M. Lefebvre, F. Legger, C. Leggett, A. Lehan, M. Lehmacher, G. Lehmann Miotto, X. Lei, W. A. Leight, A. Leisos, A. G. Leister, M. A. L. Leite, R. Leitner, D. Lellouch, B. Lemmer, K. J. C. Leney, T. Lenz, G. Lenzen, B. Lenzi, R. Leone, S. Leone, K. Leonhardt, C. Leonidopoulos, S. Leontsinis, C. Leroy, C. G. Lester, C. M. Lester, M. Levchenko, J. Levêque, D. Levin, L. J. Levinson, M. Levy, A. Lewis, G. H. Lewis, A. M. Leyko, M. Leyton, B. Li, B. Li, H. Li, H. L. Li, L. Li, L. Li, S. Li, Y. Li, Z. Liang, H. Liao, B. Liberti, P. Lichard, K. Lie, J. Liebal, W. Liebig, C. Limbach, A. Limosani, S. C. Lin, T. H. Lin, F. Linde, B. E. Lindquist, J. T. Linnemann, E. Lipeles, A. Lipniacka, M. Lisovyi, T. M. Liss, D. Lissauer, A. Lister, A. M. Litke, B. Liu, D. Liu, J. B. Liu, K. Liu, L. Liu, M. Liu, M. Liu, Y. Liu, M. Livan, S. S. A. Livermore, A. Lleres, J. Llorente Merino, S. L. Lloyd, F. Lo Sterzo, E. Lobodzinska, P. Loch, W. S. Lockman, T. Loddenkoetter, F. K. Loebinger, A. E. Loevschall-Jensen, A. Loginov, T. Lohse, K. Lohwasser, M. Lokajicek, V. P. Lombardo, B. A. Long, J. D. Long, R. E. Long, L. Lopes, D. Lopez Mateos, B. Lopez Paredes, I. Lopez Paz, J. Lorenz, N. Lorenzo Martinez, M. Losada, P. Loscutoff, X. Lou, A. Lounis, J. Love, P. A. Love, A. J. Lowe, F. Lu, N. Lu, H. J. Lubatti, C. Luci, A. Lucotte, F. Luehring, W. Lukas, L. Luminari, O. Lundberg, B. Lund-Jensen, M. Lungwitz, D. Lynn, R. Lysak, E. Lytken, H. Ma, L. L. Ma, G. Maccarrone, A. Macchiolo, J. Machado Miguens, D. Macina, D. Madaffari, R. Madar, H. J. Maddocks, W. F. Mader, A. Madsen, M. Maeno, T. Maeno, E. Magradze, K. Mahboubi, J. Mahlstedt, S. Mahmoud, C. Maiani, C. Maidantchik, A. A. Maier, A. Maio, S. Majewski, Y. Makida, N. Makovec, P. Mal, B. Malaescu, Pa. Malecki, V. P. Maleev, F. Malek, U. Mallik, D. Malon, C. Malone, S. Maltezos, V. M. Malyshev, S. Malyukov, J. Mamuzic, B. Mandelli, L. Mandelli, I. Mandić, R. Mandrysch, J. Maneira, A. Manfredini, L. Manhaes de Andrade Filho, J. A. Manjarres Ramos, A. Mann, P. M. Manning, A. Manousakis-Katsikakis, B. Mansoulie, R. Mantifel, L. Mapelli, L. March, J. F. Marchand, G. Marchiori, M. Marcisovsky, C. P. Marino, M. Marjanovic, C. N. Marques, F. Marroquim, S. P. Marsden, Z. Marshall, L. F. Marti, S. Marti-Garcia, B. Martin, B. Martin, T. A. Martin, V. J. Martin, B. Martin dit Latour, H. Martinez, M. Martinez, S. Martin-Haugh, A. C. Martyniuk, M. Marx, F. Marzano, A. Marzin, L. Masetti, T. Mashimo, R. Mashinistov, J. Masik, A. L. Maslennikov, I. Massa, L. Massa, N. Massol, P. Mastrandrea, A. Mastroberardino, T. Masubuchi, P. Mättig, J. Mattmann, J. Maurer, S. J. Maxfield, D. A. Maximov, R. Mazini, L. Mazzaferro, G. Mc Goldrick, S. P. Mc Kee, A. McCarn, R. L. McCarthy, T. G. McCarthy, N. A. McCubbin, K. W. McFarlane, J. A. Mcfayden, G. Mchedlidze, S. J. McMahon, R. A. McPherson, A. Meade, J. Mechnich, M. Medinnis, S. Meehan, S. Mehlhase, A. Mehta, K. Meier, C. Meineck, B. Meirose, C. Melachrinos, B. R. Mellado Garcia, F. Meloni, A. Mengarelli, S. Menke, E. Meoni, K. M. Mercurio, S. Mergelmeyer, N. Meric, P. Mermod, L. Merola, C. Meroni, F. S. Merritt, H. Merritt, A. Messina, J. Metcalfe, A. S. Mete, C. Meyer, C. Meyer, J.-P. Meyer, J. Meyer, R. P. Middleton, S. Migas, L. Mijović, G. Mikenberg, M. Mikestikova, M. Mikuž, A. Milic, D. W. Miller, C. Mills, A. Milov, D. A. Milstead, D. Milstein, A. A. Minaenko, I. A. Minashvili, A. I. Mincer, B. Mindur, M. Mineev, Y. Ming, L. M. Mir, G. Mirabelli, T. Mitani, J. Mitrevski, V. A. Mitsou, S. Mitsui, A. Miucci, P. S. Miyagawa, J. U. Mjörnmark, T. Moa, K. Mochizuki, S. Mohapatra, W. Mohr, S. Molander, R. Moles-Valls, K. Mönig, C. Monini, J. Monk, E. Monnier, J. Montejo Berlingen, F. Monticelli, S. Monzani, R. W. Moore, N. Morange, D. Moreno, M. Moreno Llácer, P. Morettini, M. Morgenstern, M. Morii, S. Moritz, A. K. Morley, G. Mornacchi, J. D. Morris, L. Morvaj, H. G. Moser, M. Mosidze, J. Moss, K. Motohashi, R. Mount, E. Mountricha, S. V. Mouraviev, E. J. W. Moyse, S. Muanza, R. D. Mudd, F. Mueller, J. Mueller, K. Mueller, T. Mueller, T. Mueller, D. Muenstermann, Y. Munwes, J. A. Murillo Quijada, W. J. Murray, H. Musheghyan, E. Musto, A. G. Myagkov, M. Myska, O. Nackenhorst, J. Nadal, K. Nagai, R. Nagai, Y. Nagai, K. Nagano, A. Nagarkar, Y. Nagasaka, M. Nagel, A. M. Nairz, Y. Nakahama, K. Nakamura, T. Nakamura, I. Nakano, H. Namasivayam, G. Nanava, R. Narayan, T. Nattermann, T. Naumann, G. Navarro, R. Nayyar, H. A. Neal, P. Yu. Nechaeva, T. J. Neep, P. D. Nef, A. Negri, G. Negri, M. Negrini, S. Nektarijevic, A. Nelson, T. K. Nelson, S. Nemecek, P. Nemethy, A. A. Nepomuceno, M. Nessi, M. S. Neubauer, M. Neumann, R. M. Neves, P. Nevski, P. R. Newman, D. H. Nguyen, R. B. Nickerson, R. Nicolaidou, B. Nicquevert, J. Nielsen, N. Nikiforou, A. Nikiforov, V. Nikolaenko, I. Nikolic-Audit, K. Nikolics, K. Nikolopoulos, P. Nilsson, Y. Ninomiya, A. Nisati, R. Nisius, T. Nobe, L. Nodulman, M. Nomachi, I. Nomidis, S. Norberg, M. Nordberg, O. Novgorodova, S. Nowak, M. Nozaki, L. Nozka, K. Ntekas, G. Nunes Hanninger, T. Nunnemann, E. Nurse, F. Nuti, B. J. O’Brien, F. O’grady, D. C. O’Neil, V. O’Shea, F. G. Oakham, H. Oberlack, T. Obermann, J. Ocariz, A. Ochi, M. I. Ochoa, S. Oda, S. Odaka, H. Ogren, A. Oh, S. H. Oh, C. C. Ohm, H. Ohman, W. Okamura, H. Okawa, Y. Okumura, T. Okuyama, A. Olariu, A. G. Olchevski, S. A. Olivares Pino, D. Oliveira Damazio, E. Oliver Garcia, A. Olszewski, J. Olszowska, A. Onofre, P. U. E. Onyisi, C. J. Oram, M. J. Oreglia, Y. Oren, D. Orestano, N. Orlando, C. Oropeza Barrera, R. S. Orr, B. Osculati, R. Ospanov, G. Otero y Garzon, H. Otono, M. Ouchrif, E. A. Ouellette, F. Ould-Saada, A. Ouraou, K. P. Oussoren, Q. Ouyang, A. Ovcharova, M. Owen, V. E. Ozcan, N. Ozturk, K. Pachal, A. Pacheco Pages, C. Padilla Aranda, M. Pagáčová, S. Pagan Griso, E. Paganis, C. Pahl, F. Paige, P. Pais, K. Pajchel, G. Palacino, S. Palestini, M. Palka, D. Pallin, A. Palma, J. D. Palmer, Y. B. Pan, E. Panagiotopoulou, J. G. Panduro Vazquez, P. Pani, N. Panikashvili, S. Panitkin, D. Pantea, L. Paolozzi, Th. D. Papadopoulou, K. Papageorgiou, A. Paramonov, D. Paredes Hernandez, M. A. Parker, F. Parodi, J. A. Parsons, U. Parzefall, E. Pasqualucci, S. Passaggio, A. Passeri, F. Pastore, Fr. Pastore, G. Pásztor, S. Pataraia, N. D. Patel, J. R. Pater, S. Patricelli, T. Pauly, J. Pearce, L. E. Pedersen, M. Pedersen, S. Pedraza Lopez, R. Pedro, S. V. Peleganchuk, D. Pelikan, H. Peng, B. Penning, J. Penwell, D. V. Perepelitsa, E. Perez Codina, M. T. Pérez García-Estañ, V. Perez Reale, L. Perini, H. Pernegger, R. Perrino, R. Peschke, V. D. Peshekhonov, K. Peters, R. F. Y. Peters, B. A. Petersen, T. C. Petersen, E. Petit, A. Petridis, C. Petridou, E. Petrolo, F. Petrucci, N. E. Pettersson, R. Pezoa, P. W. Phillips, G. Piacquadio, E. Pianori, A. Picazio, E. Piccaro, M. Piccinini, R. Piegaia, D. T. Pignotti, J. E. Pilcher, A. D. Pilkington, J. Pina, M. Pinamonti, A. Pinder, J. L. Pinfold, A. Pingel, B. Pinto, S. Pires, M. Pitt, C. Pizio, L. Plazak, M.-A. Pleier, V. Pleskot, E. Plotnikova, P. Plucinski, S. Poddar, F. Podlyski, R. Poettgen, L. Poggioli, D. Pohl, M. Pohl, G. Polesello, A. Policicchio, R. Polifka, A. Polini, C. S. Pollard, V. Polychronakos, K. Pommès, L. Pontecorvo, B. G. Pope, G. A. Popeneciu, D. S. Popovic, A. Poppleton, X. Portell Bueso, S. Pospisil, K. Potamianos, I. N. Potrap, C. J. Potter, C. T. Potter, G. Poulard, J. Poveda, V. Pozdnyakov, P. Pralavorio, A. Pranko, S. Prasad, R. Pravahan, S. Prell, D. Price, J. Price, L. E. Price, D. Prieur, M. Primavera, M. Proissl, K. Prokofiev, F. Prokoshin, E. Protopapadaki, S. Protopopescu, J. Proudfoot, M. Przybycien, H. Przysiezniak, E. Ptacek, D. Puddu, E. Pueschel, D. Puldon, M. Purohit, P. Puzo, J. Qian, G. Qin, Y. Qin, A. Quadt, D. R. Quarrie, W. B. Quayle, M. Queitsch-Maitland, D. Quilty, A. Qureshi, V. Radeka, V. Radescu, S. K. Radhakrishnan, P. Radloff, P. Rados, F. Ragusa, G. Rahal, S. Rajagopalan, M. Rammensee, A. S. Randle-Conde, C. Rangel-Smith, K. Rao, F. Rauscher, T. C. Rave, T. Ravenscroft, M. Raymond, A. L. Read, N. P. Readioff, D. M. Rebuzzi, A. Redelbach, G. Redlinger, R. Reece, K. Reeves, L. Rehnisch, H. Reisin, M. Relich, C. Rembser, H. Ren, Z. L. Ren, A. Renaud, M. Rescigno, S. Resconi, O. L. Rezanova, P. Reznicek, R. Rezvani, R. Richter, M. Ridel, P. Rieck, J. Rieger, M. Rijssenbeek, A. Rimoldi, L. Rinaldi, E. Ritsch, I. Riu, F. Rizatdinova, E. Rizvi, S. H. Robertson, A. Robichaud-Veronneau, D. Robinson, J. E. M. Robinson, A. Robson, C. Roda, L. Rodrigues, S. Roe, O. Røhne, S. Rolli, A. Romaniouk, M. Romano, E. Romero Adam, N. Rompotis, M. Ronzani, L. Roos, E. Ros, S. Rosati, K. Rosbach, M. Rose, P. Rose, P. L. Rosendahl, O. Rosenthal, V. Rossetti, E. Rossi, L. P. Rossi, R. Rosten, M. Rotaru, I. Roth, J. Rothberg, D. Rousseau, C. R. Royon, A. Rozanov, Y. Rozen, X. Ruan, F. Rubbo, I. Rubinskiy, V. I. Rud, C. Rudolph, M. S. Rudolph, F. Rühr, A. Ruiz-Martinez, Z. Rurikova, N. A. Rusakovich, A. Ruschke, J. P. Rutherfoord, N. Ruthmann, Y. F. Ryabov, M. Rybar, G. Rybkin, N. C. Ryder, A. F. Saavedra, S. Sacerdoti, A. Saddique, I. Sadeh, H. F.-W. Sadrozinski, R. Sadykov, F. Safai Tehrani, H. Sakamoto, Y. Sakurai, G. Salamanna, A. Salamon, M. Saleem, D. Salek, P. H. Sales De Bruin, D. Salihagic, A. Salnikov, J. Salt, D. Salvatore, F. Salvatore, A. Salvucci, A. Salzburger, D. Sampsonidis, A. Sanchez, J. Sánchez, V. Sanchez Martinez, H. Sandaker, R. L. Sandbach, H. G. Sander, M. P. Sanders, M. Sandhoff, T. Sandoval, C. Sandoval, R. Sandstroem, D. P. C. Sankey, A. Sansoni, C. Santoni, R. Santonico, H. Santos, I. Santoyo Castillo, K. Sapp, A. Sapronov, J. G. Saraiva, B. Sarrazin, G. Sartisohn, O. Sasaki, Y. Sasaki, G. Sauvage, E. Sauvan, P. Savard, D. O. Savu, C. Sawyer, L. Sawyer, D. H. Saxon, J. Saxon, C. Sbarra, A. Sbrizzi, T. Scanlon, D. A. Scannicchio, M. Scarcella, V. Scarfone, J. Schaarschmidt, P. Schacht, D. Schaefer, R. Schaefer, S. Schaepe, S. Schaetzel, U. Schäfer, A. C. Schaffer, D. Schaile, R. D. Schamberger, V. Scharf, V. A. Schegelsky, D. Scheirich, M. Schernau, M. I. Scherzer, C. Schiavi, J. Schieck, C. Schillo, M. Schioppa, S. Schlenker, E. Schmidt, K. Schmieden, C. Schmitt, S. Schmitt, B. Schneider, Y. J. Schnellbach, U. Schnoor, L. Schoeffel, A. Schoening, B. D. Schoenrock, A. L. S. Schorlemmer, M. Schott, D. Schouten, J. Schovancova, S. Schramm, M. Schreyer, C. Schroeder, N. Schuh, M. J. Schultens, H.-C. Schultz-Coulon, H. Schulz, M. Schumacher, B. A. Schumm, Ph. Schune, C. Schwanenberger, A. Schwartzman, Ph. Schwegler, Ph. Schwemling, R. Schwienhorst, J. Schwindling, T. Schwindt, M. Schwoerer, F. G. Sciacca, E. Scifo, G. Sciolla, W. G. Scott, F. Scuri, F. Scutti, J. Searcy, G. Sedov, E. Sedykh, S. C. Seidel, A. Seiden, F. Seifert, J. M. Seixas, G. Sekhniaidze, S. J. Sekula, K. E. Selbach, D. M. Seliverstov, G. Sellers, N. Semprini-Cesari, C. Serfon, L. Serin, L. Serkin, T. Serre, R. Seuster, H. Severini, T. Sfiligoj, F. Sforza, A. Sfyrla, E. Shabalina, M. Shamim, L. Y. Shan, R. Shang, J. T. Shank, M. Shapiro, P. B. Shatalov, K. Shaw, C. Y. Shehu, P. Sherwood, L. Shi, S. Shimizu, C. O. Shimmin, M. Shimojima, M. Shiyakova, A. Shmeleva, M. J. Shochet, D. Short, S. Shrestha, E. Shulga, M. A. Shupe, S. Shushkevich, P. Sicho, O. Sidiropoulou, D. Sidorov, A. Sidoti, F. Siegert, Dj. Sijacki, J. Silva, Y. Silver, D. Silverstein, S. B. Silverstein, V. Simak, O. Simard, Lj. Simic, S. Simion, E. Simioni, B. Simmons, R. Simoniello, M. Simonyan, P. Sinervo, N. B. Sinev, V. Sipica, G. Siragusa, A. Sircar, A. N. Sisakyan, S. Yu. Sivoklokov, J. Sjölin, T. B. Sjursen, H. P. Skottowe, K. Yu. Skovpen, P. Skubic, M. Slater, T. Slavicek, K. Sliwa, V. Smakhtin, B. H. Smart, L. Smestad, S. Yu. Smirnov, Y. Smirnov, L. N. Smirnova, O. Smirnova, K. M. Smith, M. Smizanska, K. Smolek, A. A. Snesarev, G. Snidero, S. Snyder, R. Sobie, F. Socher, A. Soffer, D. A. Soh, C. A. Solans, M. Solar, J. Solc, E. Yu. Soldatov, U. Soldevila, A. A. Solodkov, A. Soloshenko, O. V. Solovyanov, V. Solovyev, P. Sommer, H. Y. Song, N. Soni, A. Sood, A. Sopczak, B. Sopko, V. Sopko, V. Sorin, M. Sosebee, R. Soualah, P. Soueid, A. M. Soukharev, D. South, S. Spagnolo, F. Spanò, W. R. Spearman, F. Spettel, R. Spighi, G. Spigo, M. Spousta, T. Spreitzer, B. Spurlock, R. D. St. Denis, S. Staerz, J. Stahlman, R. Stamen, S. Stamm, E. Stanecka, R. W. Stanek, C. Stanescu, M. Stanescu-Bellu, M. M. Stanitzki, S. Stapnes, E. A. Starchenko, J. Stark, P. Staroba, P. Starovoitov, R. Staszewski, P. Stavina, P. Steinberg, B. Stelzer, H. J. Stelzer, O. Stelzer-Chilton, H. Stenzel, S. Stern, G. A. Stewart, J. A. Stillings, M. C. Stockton, M. Stoebe, G. Stoicea, P. Stolte, S. Stonjek, A. R. Stradling, A. Straessner, M. E. Stramaglia, J. Strandberg, S. Strandberg, A. Strandlie, E. Strauss, M. Strauss, P. Strizenec, R. Ströhmer, D. M. Strom, R. Stroynowski, S. A. Stucci, B. Stugu, N. A. Styles, D. Su, J. Su, R. Subramaniam, A. Succurro, Y. Sugaya, C. Suhr, M. Suk, V. V. Sulin, S. Sultansoy, T. Sumida, S. Sun, X. Sun, J. E. Sundermann, K. Suruliz, G. Susinno, M. R. Sutton, Y. Suzuki, M. Svatos, S. Swedish, M. Swiatlowski, I. Sykora, T. Sykora, D. Ta, C. Taccini, K. Tackmann, J. Taenzer, A. Taffard, R. Tafirout, N. Taiblum, H. Takai, R. Takashima, H. Takeda, T. Takeshita, Y. Takubo, M. Talby, A. A. Talyshev, J. Y. C. Tam, K. G. Tan, J. Tanaka, R. Tanaka, S. Tanaka, S. Tanaka, A. J. Tanasijczuk, B. B. Tannenwald, N. Tannoury, S. Tapprogge, S. Tarem, F. Tarrade, G. F. Tartarelli, P. Tas, M. Tasevsky, T. Tashiro, E. Tassi, A. Tavares Delgado, Y. Tayalati, F. E. Taylor, G. N. Taylor, W. Taylor, F. A. Teischinger, M. Teixeira Dias Castanheira, P. Teixeira-Dias, K. K. Temming, H. Ten Kate, P. K. Teng, J. J. Teoh, S. Terada, K. Terashi, J. Terron, S. Terzo, M. Testa, R. J. Teuscher, J. Therhaag, T. Theveneaux-Pelzer, J. P. Thomas, J. Thomas-Wilsker, E. N. Thompson, P. D. Thompson, P. D. Thompson, A. S. Thompson, L. A. Thomsen, E. Thomson, M. Thomson, W. M. Thong, R. P. Thun, F. Tian, M. J. Tibbetts, V. O. Tikhomirov, Yu. A. Tikhonov, S. Timoshenko, E. Tiouchichine, P. Tipton, S. Tisserant, T. Todorov, S. Todorova-Nova, B. Toggerson, J. Tojo, S. Tokár, K. Tokushuku, K. Tollefson, L. Tomlinson, M. Tomoto, L. Tompkins, K. Toms, N. D. Topilin, E. Torrence, H. Torres, E. Torró Pastor, J. Toth, F. Touchard, D. R. Tovey, H. L. Tran, T. Trefzger, L. Tremblet, A. Tricoli, I. M. Trigger, S. Trincaz-Duvoid, M. F. Tripiana, W. Trischuk, B. Trocmé, C. Troncon, M. Trottier-McDonald, M. Trovatelli, P. True, M. Trzebinski, A. Trzupek, C. Tsarouchas, J. C.-L. Tseng, P. V. Tsiareshka, D. Tsionou, G. Tsipolitis, N. Tsirintanis, S. Tsiskaridze, V. Tsiskaridze, E. G. Tskhadadze, I. I. Tsukerman, V. Tsulaia, S. Tsuno, D. Tsybychev, A. Tudorache, V. Tudorache, A. N. Tuna, S. A. Tupputi, S. Turchikhin, D. Turecek, I. Turk Cakir, R. Turra, P. M. Tuts, A. Tykhonov, M. Tylmad, M. Tyndel, K. Uchida, I. Ueda, R. Ueno, M. Ughetto, M. Ugland, M. Uhlenbrock, F. Ukegawa, G. Unal, A. Undrus, G. Unel, F. C. Ungaro, Y. Unno, C. Unverdorben, D. Urbaniec, P. Urquijo, G. Usai, A. Usanova, L. Vacavant, V. Vacek, B. Vachon, N. Valencic, S. Valentinetti, A. Valero, L. Valery, S. Valkar, E. Valladolid Gallego, S. Vallecorsa, J. A. Valls Ferrer, W. Van Den Wollenberg, P. C. Van Der Deijl, R. van der Geer, H. van der Graaf, R. Van Der Leeuw, D. van der Ster, N. van Eldik, P. van Gemmeren, J. Van Nieuwkoop, I. van Vulpen, M. C. van Woerden, M. Vanadia, W. Vandelli, R. Vanguri, A. Vaniachine, P. Vankov, F. Vannucci, G. Vardanyan, R. Vari, E. W. Varnes, T. Varol, D. Varouchas, A. Vartapetian, K. E. Varvell, F. Vazeille, T. Vazquez Schroeder, J. Veatch, F. Veloso, S. Veneziano, A. Ventura, D. Ventura, M. Venturi, N. Venturi, A. Venturini, V. Vercesi, M. Verducci, W. Verkerke, J. C. Vermeulen, A. Vest, M. C. Vetterli, O. Viazlo, I. Vichou, T. Vickey, O. E. Vickey Boeriu, G. H. A. Viehhauser, S. Viel, R. Vigne, M. Villa, M. Villaplana Perez, E. Vilucchi, M. G. Vincter, V. B. Vinogradov, J. Virzi, I. Vivarelli, F. Vives Vaque, S. Vlachos, D. Vladoiu, M. Vlasak, A. Vogel, M. Vogel, P. Vokac, G. Volpi, M. Volpi, H. von der Schmitt, H. von Radziewski, E. von Toerne, V. Vorobel, K. Vorobev, M. Vos, R. Voss, J. H. Vossebeld, N. Vranjes, M. Vranjes Milosavljevic, V. Vrba, M. Vreeswijk, T. Vu Anh, R. Vuillermet, I. Vukotic, Z. Vykydal, P. Wagner, W. Wagner, H. Wahlberg, S. Wahrmund, J. Wakabayashi, J. Walder, R. Walker, W. Walkowiak, R. Wall, P. Waller, B. Walsh, C. Wang, C. Wang, F. Wang, H. Wang, H. Wang, J. Wang, J. Wang, K. Wang, R. Wang, S. M. Wang, T. Wang, X. Wang, C. Wanotayaroj, A. Warburton, C. P. Ward, D. R. Wardrope, M. Warsinsky, A. Washbrook, C. Wasicki, P. M. Watkins, A. T. Watson, I. J. Watson, M. F. Watson, G. Watts, S. Watts, B. M. Waugh, S. Webb, M. S. Weber, S. W. Weber, J. S. Webster, A. R. Weidberg, P. Weigell, B. Weinert, J. Weingarten, C. Weiser, H. Weits, P. S. Wells, T. Wenaus, D. Wendland, Z. Weng, T. Wengler, S. Wenig, N. Wermes, M. Werner, P. Werner, M. Wessels, J. Wetter, K. Whalen, A. White, M. J. White, R. White, S. White, D. Whiteson, D. Wicke, F. J. Wickens, W. Wiedenmann, M. Wielers, P. Wienemann, C. Wiglesworth, L. A. M. Wiik-Fuchs, P. A. Wijeratne, A. Wildauer, M. A. Wildt, H. G. Wilkens, J. Z. Will, H. H. Williams, S. Williams, C. Willis, S. Willocq, A. Wilson, J. A. Wilson, I. Wingerter-Seez, F. Winklmeier, B. T. Winter, M. Wittgen, T. Wittig, J. Wittkowski, S. J. Wollstadt, M. W. Wolter, H. Wolters, B. K. Wosiek, J. Wotschack, M. J. Woudstra, K. W. Wozniak, M. Wright, M. Wu, S. L. Wu, X. Wu, Y. Wu, E. Wulf, T. R. Wyatt, B. M. Wynne, S. Xella, M. Xiao, D. Xu, L. Xu, B. Yabsley, S. Yacoob, R. Yakabe, M. Yamada, H. Yamaguchi, Y. Yamaguchi, A. Yamamoto, K. Yamamoto, S. Yamamoto, T. Yamamura, T. Yamanaka, K. Yamauchi, Y. Yamazaki, Z. Yan, H. Yang, H. Yang, U. K. Yang, Y. Yang, S. Yanush, L. Yao, W.-M. Yao, Y. Yasu, E. Yatsenko, K. H. Yau Wong, J. Ye, S. Ye, A. L. Yen, E. Yildirim, M. Yilmaz, R. Yoosoofmiya, K. Yorita, R. Yoshida, K. Yoshihara, C. Young, C. J. S. Young, S. Youssef, D. R. Yu, J. Yu, J. M. Yu, J. Yu, L. Yuan, A. Yurkewicz, I. Yusuff, B. Zabinski, R. Zaidan, A. M. Zaitsev, A. Zaman, S. Zambito, L. Zanello, D. Zanzi, C. Zeitnitz, M. Zeman, A. Zemla, K. Zengel, O. Zenin, T. Ženiš, D. Zerwas, G. Zevi della Porta, D. Zhang, F. Zhang, H. Zhang, J. Zhang, L. Zhang, X. Zhang, Z. Zhang, Z. Zhao, A. Zhemchugov, J. Zhong, B. Zhou, L. Zhou, N. Zhou, C. G. Zhu, H. Zhu, J. Zhu, Y. Zhu, X. Zhuang, K. Zhukov, A. Zibell, D. Zieminska, N. I. Zimine, C. Zimmermann, R. Zimmermann, S. Zimmermann, S. Zimmermann, Z. Zinonos, M. Ziolkowski, G. Zobernig, A. Zoccoli, M. zur Nedden, G. Zurzolo, V. Zutshi, L. Zwalinski

**Affiliations:** 1 Department of Physics, University of Adelaide, Adelaide, Australia; 2 Physics Department, SUNY Albany, Albany, NY USA; 3 Department of Physics, University of Alberta, Edmonton, AB Canada; 4 Department of Physics, Ankara University, Ankara, Turkey; Department of Physics, Gazi University, Ankara, Turkey; Division of Physics, TOBB University of Economics and Technology, Ankara, Turkey; Turkish Atomic Energy Authority, Ankara, Turkey; 5 LAPP, CNRS/IN2P3 and Université de Savoie, Annecy-le-Vieux, France; 6 High Energy Physics Division, Argonne National Laboratory, Argonne, IL USA; 7 Department of Physics, University of Arizona, Tucson, AZ USA; 8 Department of Physics, The University of Texas at Arlington, Arlington, TX USA; 9 Physics Department, University of Athens, Athens, Greece; 10 Physics Department, National Technical University of Athens, Zografou, Greece; 11 Institute of Physics, Azerbaijan Academy of Sciences, Baku, Azerbaijan; 12 Institut de Física d’Altes Energies and Departament de Física de la Universitat Autònoma de Barcelona, Barcelona, Spain; 13 Institute of Physics, University of Belgrade, Belgrade, Serbia; Vinca Institute of Nuclear Sciences, University of Belgrade, Belgrade, Serbia; 14 Department for Physics and Technology, University of Bergen, Bergen, Norway; 15 Physics Division, Lawrence Berkeley National Laboratory and University of California, Berkeley, CA USA; 16 Department of Physics, Humboldt University, Berlin, Germany; 17 Albert Einstein Center for Fundamental Physics and Laboratory for High Energy Physics, University of Bern, Bern, Switzerland; 18 School of Physics and Astronomy, University of Birmingham, Birmingham, UK; 19 Department of Physics, Bogazici University, Istanbul, Turkey; Department of Physics, Dogus University, Istanbul, Turkey; Department of Physics Engineering, Gaziantep University, Gaziantep, Turkey; 20 INFN Sezione di Bologna, Bologna, Italy; Dipartimento di Fisica e Astronomia, Università di Bologna, Bologna, Italy; 21 Physikalisches Institut, University of Bonn, Bonn, Germany; 22 Department of Physics, Boston University, Boston, MA USA; 23 Department of Physics, Brandeis University, Waltham, MA USA; 24 Universidade Federal do Rio De Janeiro COPPE/EE/IF, Rio de Janeiro, Brazil; Federal University of Juiz de Fora (UFJF), Juiz de Fora, Brazil; Federal University of Sao Joao del Rei (UFSJ), Sao Joao del Rei, Brazil; Instituto de Fisica, Universidade de Sao Paulo, São Paulo, Brazil; 25 Physics Department, Brookhaven National Laboratory, Upton, NY USA; 26 National Institute of Physics and Nuclear Engineering, Bucharest, Romania; Physics Department, National Institute for Research and Development of Isotopic and Molecular Technologies, Cluj Napoca, Romania; University Politehnica Bucharest, Bucharest, Romania; West University in Timisoara, Timisoara, Romania; 27 Departamento de Física, Universidad de Buenos Aires, Buenos Aires, Argentina; 28 Cavendish Laboratory, University of Cambridge, Cambridge, UK; 29 Department of Physics, Carleton University, Ottawa, ON Canada; 30 CERN, Geneva, Switzerland; 31 Enrico Fermi Institute, University of Chicago, Chicago, IL USA; 32 Departamento de Física, Pontificia Universidad Católica de Chile, Santiago, Chile; Departamento de Física, Universidad Técnica Federico Santa María, Valparaiso, Chile; 33 Institute of High Energy Physics, Chinese Academy of Sciences, Beijing, China; Department of Modern Physics, University of Science and Technology of China, Hefei, Anhui, China; Department of Physics, Nanjing University, Nanjing, Jiangsu, China; School of Physics, Shandong University, Jinan, Shandong, China; Physics Department, Shanghai Jiao Tong University, Shanghai, China; 34 Laboratoire de Physique Corpusculaire, Clermont Université and Université Blaise Pascal and CNRS/IN2P3, Clermont-Ferrand, France; 35 Nevis Laboratory, Columbia University, Irvington, NY USA; 36 Niels Bohr Institute, University of Copenhagen, Copenhagen, Denmark; 37 INFN Gruppo Collegato di Cosenza, Laboratori Nazionali di Frascati, Frascati, Italy; Dipartimento di Fisica, Università della Calabria, Rende, Italy; 38 Faculty of Physics and Applied Computer Science, AGH University of Science and Technology, Kraków, Poland; Marian Smoluchowski Institute of Physics, Jagiellonian University, Kraków, Poland; 39 The Henryk Niewodniczanski Institute of Nuclear Physics, Polish Academy of Sciences, Kraków, Poland; 40 Physics Department, Southern Methodist University, Dallas, TX USA; 41 Physics Department, University of Texas at Dallas, Richardson, TX USA; 42 DESY, Hamburg and Zeuthen, Germany; 43 Institut für Experimentelle Physik IV, Technische Universität Dortmund, Dortmund, Germany; 44 Institut für Kern- und Teilchenphysik, Technische Universität Dresden, Dresden, Germany; 45 Department of Physics, Duke University, Durham, NC USA; 46 SUPA-School of Physics and Astronomy, University of Edinburgh, Edinburgh, UK; 47 INFN Laboratori Nazionali di Frascati, Frascati, Italy; 48 Fakultät für Mathematik und Physik, Albert-Ludwigs-Universität, Freiburg, Germany; 49 Section de Physique, Université de Genève, Geneva, Switzerland; 50 INFN Sezione di Genova, Genoa, Italy; Dipartimento di Fisica, Università di Genova, Genova, Italy; 51 E. Andronikashvili Institute of Physics, Iv. Javakhishvili Tbilisi State University, Tbilisi, Georgia; High Energy Physics Institute, Tbilisi State University, Tbilisi, Georgia; 52 II Physikalisches Institut, Justus-Liebig-Universität Giessen, Giessen, Germany; 53 SUPA-School of Physics and Astronomy, University of Glasgow, Glasgow, UK; 54 II Physikalisches Institut, Georg-August-Universität, Göttingen, Germany; 55 Laboratoire de Physique Subatomique et de Cosmologie, Université Grenoble-Alpes, CNRS/IN2P3, Grenoble, France; 56 Department of Physics, Hampton University, Hampton, VA USA; 57 Laboratory for Particle Physics and Cosmology, Harvard University, Cambridge, MA USA; 58 Kirchhoff-Institut für Physik, Ruprecht-Karls-Universität Heidelberg, Heidelberg, Germany; Physikalisches Institut, Ruprecht-Karls-Universität Heidelberg, Heidelberg, Germany; ZITI Institut für technische Informatik, Ruprecht-Karls-Universität Heidelberg, Mannheim, Germany; 59 Faculty of Applied Information Science, Hiroshima Institute of Technology, Hiroshima, Japan; 60 Department of Physics, Indiana University, Bloomington, IN USA; 61 Institut für Astro- und Teilchenphysik, Leopold-Franzens-Universität, Innsbruck, Austria; 62 University of Iowa, Iowa City, IA USA; 63 Department of Physics and Astronomy, Iowa State University, Ames, IA USA; 64 Joint Institute for Nuclear Research, JINR Dubna, Dubna, Russia; 65 KEK, High Energy Accelerator Research Organization, Tsukuba, Japan; 66 Graduate School of Science, Kobe University, Kobe, Japan; 67 Faculty of Science, Kyoto University, Kyoto, Japan; 68 Kyoto University of Education, Kyoto, Japan; 69 Department of Physics, Kyushu University, Fukuoka, Japan; 70 Instituto de Física La Plata, Universidad Nacional de La Plata and CONICET, La Plata, Argentina; 71 Physics Department, Lancaster University, Lancaster, UK; 72 INFN Sezione di Lecce, Lecce, Italy; Dipartimento di Matematica e Fisica, Università del Salento, Lecce, Italy; 73 Oliver Lodge Laboratory, University of Liverpool, Liverpool, UK; 74 Department of Physics, Jožef Stefan Institute and University of Ljubljana, Ljubljana, Slovenia; 75 School of Physics and Astronomy, Queen Mary University of London, London, UK; 76 Department of Physics, Royal Holloway University of London, Surrey, UK; 77 Department of Physics and Astronomy, University College London, London, UK; 78 Louisiana Tech University, Ruston, LA USA; 79 Laboratoire de Physique Nucléaire et de Hautes Energies, UPMC and Université Paris-Diderot and CNRS/IN2P3, Paris, France; 80 Fysiska institutionen, Lunds universitet, Lund, Sweden; 81 Departamento de Fisica Teorica C-15, Universidad Autonoma de Madrid, Madrid, Spain; 82 Institut für Physik, Universität Mainz, Mainz, Germany; 83 School of Physics and Astronomy, University of Manchester, Manchester, UK; 84 CPPM, Aix-Marseille Université and CNRS/IN2P3, Marseille, France; 85 Department of Physics, University of Massachusetts, Amherst, MA USA; 86 Department of Physics, McGill University, Montreal, QC Canada; 87 School of Physics, University of Melbourne, Parkville, VIC Australia; 88 Department of Physics, The University of Michigan, Ann Arbor, MI USA; 89 Department of Physics and Astronomy, Michigan State University, East Lansing, MI USA; 90 INFN Sezione di Milano, Milan, Italy; Dipartimento di Fisica, Università di Milano, Milan, Italy; 91 B.I. Stepanov Institute of Physics, National Academy of Sciences of Belarus, Minsk, Republic of Belarus; 92 National Scientific and Educational Centre for Particle and High Energy Physics, Minsk, Republic of Belarus; 93 Department of Physics, Massachusetts Institute of Technology, Cambridge, MA USA; 94 Group of Particle Physics, University of Montreal, Montreal, QC Canada; 95 P.N. Lebedev Institute of Physics, Academy of Sciences, Moscow, Russia; 96 Institute for Theoretical and Experimental Physics (ITEP), Moscow, Russia; 97 Moscow Engineering and Physics Institute (MEPhI), Moscow, Russia; 98 D.V. Skobeltsyn Institute of Nuclear Physics, M.V. Lomonosov Moscow State University, Moscow, Russia; 99 Fakultät für Physik, Ludwig-Maximilians-Universität München, Munich, Germany; 100 Max-Planck-Institut für Physik (Werner-Heisenberg-Institut), Munich, Germany; 101 Nagasaki Institute of Applied Science, Nagasaki, Japan; 102 Graduate School of Science and Kobayashi-Maskawa Institute, Nagoya University, Nagoya, Japan; 103 INFN Sezione di Napoli, Naples, Italy; Dipartimento di Fisica, Università di Napoli, Naples, Italy; 104 Department of Physics and Astronomy, University of New Mexico, Albuquerque, NM USA; 105 Institute for Mathematics, Astrophysics and Particle Physics, Radboud University Nijmegen/Nikhef, Nijmegen, The Netherlands; 106 Nikhef National Institute for Subatomic Physics and University of Amsterdam, Amsterdam, The Netherlands; 107 Department of Physics, Northern Illinois University, DeKalb, IL USA; 108 Budker Institute of Nuclear Physics, SB RAS, Novosibirsk, Russia; 109 Department of Physics, New York University, New York, NY USA; 110 Ohio State University, Columbus, OH USA; 111 Faculty of Science, Okayama University, Okayama, Japan; 112 Homer L. Dodge Department of Physics and Astronomy, University of Oklahoma, Norman, OK USA; 113 Department of Physics, Oklahoma State University, Stillwater, OK USA; 114 Palacký University, RCPTM, Olomouc, Czech Republic; 115 Center for High Energy Physics, University of Oregon, Eugene, OR USA; 116 LAL, Université Paris-Sud and CNRS/IN2P3, Orsay, France; 117 Graduate School of Science, Osaka University, Osaka, Japan; 118 Department of Physics, University of Oslo, Oslo, Norway; 119 Department of Physics, Oxford University, Oxford, UK; 120 INFN Sezione di Pavia, Pavia, Italy; Dipartimento di Fisica, Università di Pavia, Pavia, Italy; 121 Department of Physics, University of Pennsylvania, Philadelphia, PA USA; 122 Petersburg Nuclear Physics Institute, Gatchina, Russia; 123 INFN Sezione di Pisa, Pisa, Italy; Dipartimento di Fisica E. Fermi, Università di Pisa, Pisa, Italy; 124 Department of Physics and Astronomy, University of Pittsburgh, Pittsburgh, PA USA; 125 Laboratorio de Instrumentacao e Fisica Experimental de Particulas-LIP, Lisbon, Portugal; Faculdade de Ciências, Universidade de Lisboa, Lisbon, Portugal; Department of Physics, University of Coimbra, Coimbra, Portugal; Centro de Física Nuclear da Universidade de Lisboa, Lisbon, Portugal; Departamento de Fisica, Universidade do Minho, Braga, Portugal; Departamento de Fisica Teorica y del Cosmos and CAFPE, Universidad de Granada, Granada, Spain; Dep Fisica and CEFITEC of Faculdade de Ciencias e Tecnologia, Universidade Nova de Lisboa, Caparica, Portugal; 126 Institute of Physics, Academy of Sciences of the Czech Republic, Prague, Czech Republic; 127 Czech Technical University in Prague, Prague, Czech Republic; 128 Faculty of Mathematics and Physics, Charles University in Prague, Prague, Czech Republic; 129 State Research Center Institute for High Energy Physics, Protvino, Russia; 130 Particle Physics Department, Rutherford Appleton Laboratory, Didcot, UK; 131 Physics Department, University of Regina, Regina, SK Canada; 132 Ritsumeikan University, Kusatsu, Shiga Japan; 133 INFN Sezione di Roma, Rome, Italy; Dipartimento di Fisica, Sapienza Università di Roma, Rome, Italy; 134 INFN Sezione di Roma Tor Vergata, Rome, Italy; Dipartimento di Fisica, Università di Roma Tor Vergata, Rome, Italy; 135 INFN Sezione di Roma Tre, Rome, Italy; Dipartimento di Matematica e Fisica, Università Roma Tre, Rome, Italy; 136 Faculté des Sciences Ain Chock, Réseau Universitaire de Physique des Hautes Energies-Université Hassan II, Casablanca, Morocco; Centre National de l’Energie des Sciences Techniques Nucleaires, Rabat, Morocco; Faculté des Sciences Semlalia, Université Cadi Ayyad, LPHEA-Marrakech, Marrakech, Morocco; Faculté des Sciences, Université Mohamed Premier and LPTPM, Oujda, Morocco; Faculté des Sciences, Université Mohammed V-Agdal, Rabat, Morocco; 137 DSM/IRFU (Institut de Recherches sur les Lois Fondamentales de l’Univers), CEA Saclay (Commissariat à l’Energie Atomique et aux Energies Alternatives), Gif-sur-Yvette, France; 138 Santa Cruz Institute for Particle Physics, University of California Santa Cruz, Santa Cruz, CA USA; 139 Department of Physics, University of Washington, Seattle, WA USA; 140 Department of Physics and Astronomy, University of Sheffield, Sheffield, UK; 141 Department of Physics, Shinshu University, Nagano, Japan; 142 Fachbereich Physik, Universität Siegen, Siegen, Germany; 143 Department of Physics, Simon Fraser University, Burnaby, BC Canada; 144 SLAC National Accelerator Laboratory, Stanford, CA USA; 145 Faculty of Mathematics, Physics and Informatics, Comenius University, Bratislava, Slovak Republic; Department of Subnuclear Physics, Institute of Experimental Physics of the Slovak Academy of Sciences, Kosice, Slovak Republic; 146 Department of Physics, University of Cape Town, Cape Town, South Africa; Department of Physics, University of Johannesburg, Johannesburg, South Africa; School of Physics, University of the Witwatersrand, Johannesburg, South Africa; 147 Department of Physics, Stockholm University, Stockholm, Sweden; The Oskar Klein Centre, Stockholm, Sweden; 148 Physics Department, Royal Institute of Technology, Stockholm, Sweden; 149 Departments of Physics and Astronomy and Chemistry, Stony Brook University, Stony Brook, NY USA; 150 Department of Physics and Astronomy, University of Sussex, Brighton, UK; 151 School of Physics, University of Sydney, Sydney, Australia; 152 Institute of Physics, Academia Sinica, Taipei, Taiwan; 153 Department of Physics, Technion: Israel Institute of Technology, Haifa, Israel; 154 Raymond and Beverly Sackler School of Physics and Astronomy, Tel Aviv University, Tel Aviv, Israel; 155 Department of Physics, Aristotle University of Thessaloniki, Thessaloniki, Greece; 156 International Center for Elementary Particle Physics and Department of Physics, The University of Tokyo, Tokyo, Japan; 157 Graduate School of Science and Technology, Tokyo Metropolitan University, Tokyo, Japan; 158 Department of Physics, Tokyo Institute of Technology, Tokyo, Japan; 159 Department of Physics, University of Toronto, Toronto, ON Canada; 160 TRIUMF, Vancouver, BC, Canada; Department of Physics and Astronomy, York University, Toronto, ON Canada; 161 Faculty of Pure and Applied Sciences, University of Tsukuba, Tsukuba, Japan; 162 Department of Physics and Astronomy, Tufts University, Medford, MA USA; 163 Centro de Investigaciones, Universidad Antonio Narino, Bogota, Colombia; 164 Department of Physics and Astronomy, University of California Irvine, Irvine, CA USA; 165 INFN Gruppo Collegato di Udine, Sezione di Trieste, Udine, Italy; ICTP, Trieste, Italy; Dipartimento di Chimica, Fisica e Ambiente, Università di Udine, Udine, Italy; 166 Department of Physics, University of Illinois, Urbana, IL USA; 167 Department of Physics and Astronomy, University of Uppsala, Uppsala, Sweden; 168 Instituto de Física Corpuscular (IFIC) and Departamento de Física Atómica, Molecular y Nuclear and Departamento de Ingeniería Electrónica and Instituto de Microelectrónica de Barcelona (IMB-CNM), University of Valencia and CSIC, Valencia, Spain; 169 Department of Physics, University of British Columbia, Vancouver, BC Canada; 170 Department of Physics and Astronomy, University of Victoria, Victoria, BC Canada; 171 Department of Physics, University of Warwick, Coventry, UK; 172 Waseda University, Tokyo, Japan; 173 Department of Particle Physics, The Weizmann Institute of Science, Rehovot, Israel; 174 Department of Physics, University of Wisconsin, Madison, WI USA; 175 Fakultät für Physik und Astronomie, Julius-Maximilians-Universität, Würzburg, Germany; 176 Fachbereich C Physik, Bergische Universität Wuppertal, Wuppertal, Germany; 177 Department of Physics, Yale University, New Haven, CT USA; 178 Yerevan Physics Institute, Yerevan, Armenia; 179 Centre de Calcul de l’Institut National de Physique Nucléaire et de Physique des Particules (IN2P3), Villeurbanne, France; 180 CERN, 1211 Geneva 23, Switzerland

## Abstract

Additional jet activity in dijet events is measured using ${pp}$ collisions at ATLAS at a centre-of-mass energy of $7\,\mathrm{TeV}$, for jets reconstructed using the ${\mathrm{anti}\hbox {-}{k_t}}$ algorithm with radius parameter ${R=0.6}$. This is done using variables such as the fraction of dijet events without an additional jet in the rapidity interval bounded by the dijet subsystem and correlations between the azimuthal angles of the dijet s. They are presented, both with and without a veto on additional jet activity in the rapidity interval, as a function of the scalar average of the transverse momenta of the dijet s and of the rapidity interval size. The double differential dijet cross section is also measured as a function of the interval size and the azimuthal angle between the dijet s. These variables probe differences in the approach to resummation of large logarithms when performing QCD calculations. The data are compared to powheg, interfaced to the pythia 8 and herwig parton shower generators, as well as to hej with and without interfacing it to the ariadne parton shower generator. None of the theoretical predictions agree with the data across the full phase-space considered; however, powheg+pythia 8 and hej+ariadne are found to provide the best agreement with the data. These measurements use the full data sample collected with the ATLAS detector in $7\,\mathrm{TeV}$ ${pp}$ collisions at the LHC and correspond to integrated luminosities of $36.1\,\mathrm{pb}^{-1}$ and $4.5\,\mathrm{fb}^{-1}$ for data collected during 2010 and 2011, respectively.

## Introduction

The large hadron collider (LHC) has opened up a new kinematic regime to test perturbative QCD (pQCD) using measurements of jet production. Next-to-leading-order QCD predictions for inclusive jet and dijet cross section s have been found to describe the data at the highest measured energies [1–7]. However, purely fixed-order calculations are expected to describe the data poorly wherever higher-order corrections to a given observable are important. In such cases, higher orders in perturbation theory must be resummed; this resummation is typically performed in terms of ${\ln (1/ x)}$, where ${x}$ is Bjorken-$x$, the Balitsky–Fadin–Kuraev–Lipatov (BFKL) approach [8–11], or in terms of ${\ln (Q^2)}$, where ${Q^2}$ is the virtuality of the interaction, the Dokshitzer–Gribov–Lipatov–Altarelli–Parisi (DGLAP) approach [12–14]. These resummations provide approximations that are most valid in phase-space regions for which the resummed terms provide a dominant contribution to the observable. Such a situation exists in dijet topologies when the two jets have a large rapidity separation or when a veto is applied to additional jet activity in the rapidity interval bounded by the dijet system [15]. In these regions of phase-space, higher order corrections proportional to the rapidity separation and the logarithm of the scalar average of the transverse momenta of the dijets become increasing important: these must be summed to all orders to obtain accurate theoretical predictions.

When studying these phase-space regions, a particularly interesting observable is the gap fraction, ${f\left( {Q_{0}} \right) }$, defined as ${f\left( {Q_{0}} \right) = \sigma _{\mathrm {jj}}\left( {Q_{0}} \right) /\sigma _{\mathrm {jj}}}$ where ${\sigma _{\mathrm {jj}}}$ is the inclusive dijet cross section and ${\sigma _{\mathrm {jj}}\left( {Q_{0}} \right) }$ is the cross section for dijet production in the absence of jets with transverse momentum greater than ${Q_{0}}$ in the rapidity interval bounded by the dijet system. The variable ${Q_{0}}$ is referred to as the veto scale. In the limit of large rapidity separation, ${\Delta y}$, between the jet centroids, the gap fraction is expected to be sensitive to BFKL dynamics [16, 17]. Alternatively, when the scalar average of the transverse momenta of the dijet s, ${\overline{p_{\mathrm{T}}}}$, is much larger than the veto scale, the effects of wide-angle soft gluon radiation may become important [18–20]. Finally, dijet production via ${t}$-channel colour-singlet exchange [21] is expected to provide an increasingly important contribution to the total dijet cross section when both of these limits are approached simultaneously. The mean number of jets above the veto scale in the rapidity interval between the dijet s is presented as an alternative measurement of hard jet emissions in the rapidity interval.

A complementary probe of higher-order QCD effects can be made by studying the azimuthal angle between the jets in the dijet system, ${\Delta \phi }$. A purely ${2\rightarrow 2}$ partonic scatter produces final-state partons back-to-back in azimuthal angle. Any additional quark or gluon emission alters the balance between the partons and produces an azimuthal decorrelation, the predicted magnitude of which is different for fixed-order calculations, BFKL-inspired resummations and DGLAP-inspired resummations [22]. In particular, the azimuthal decorrelation is expected to increase with increasing rapidity separation if BFKL effects are present [23, 24]. To discriminate between DGLAP-like and BFKL-like behaviour, the azimuthal angular moments ${\langle \cos \left( n \left( \pi - \Delta \phi \right) \right) \rangle }$ where ${n}$ is an integer and the angled brackets indicate the profiled mean over all events, have been proposed [23–25]. In addition, taking the ratio of different angular moments is predicted to enhance BFKL effects [24, 26, 27].

Previous measurements have been made of dijet production for which a strict veto, of order of ${\Lambda _\text {QCD}}$, was imposed on the emission of additional radiation in the inter-jet region, so-called “rapidity gap” events, at HERA [28–30] and at the Tevatron [31–35]. At the LHC, measurements of forward rapidity gaps and dijet production have been made by ATLAS [36, 37], while ratios of exclusive-to-inclusive dijet cross section s have been measured at CMS [38]. Azimuthal decorrelations for central dijet s have also been measured at the LHC by ATLAS [39] and CMS [40] and before that by D0 at the Tevatron [41]. Finally, a previous study of azimuthal angular decorrelations for widely separated dijet s was made by D0 at the Tevatron [42].

This paper presents measurements of the gap fraction and the mean number of jets in the rapidity interval as functions of both the dijet rapidity separation and the scalar average of the transverse momenta of the dijet s. Measurements of the first azimuthal angular moment, the ratio of the first two moments and the double-differential cross section s as functions of ${\Delta \phi }$ and ${\Delta y}$ are also presented, both for an inclusive dijet sample and for events where a jet veto is imposed. Previous results are extended out to a dijet rapidity separation of ${{\Delta y} = 8}$ as well as to dijet transverse momenta up to ${{\overline{p_{\mathrm{T}}}} = 1.5\,\mathrm{TeV}}$, the effective kinematic limits of the ATLAS detector for ${pp}$ collisions at a centre-of-mass energy, ${\sqrt{s}=7\,\mathrm{TeV}}$. The measurements are obtained using the full ${pp}$ collision datasets recorded during 2010 and 2011, corresponding to integrated luminosities of ${36.1\pm 1.3}\,\mathrm{pb}^{-1}$ and ${4.5\pm 0.1}\,\mathrm{fb}^{-1}$, respectively [43]. The two datasets are used in complementary areas of phase-space: the data collected during 2010 are used in the full rapidity range covered by the detector, probing large rapidity separations, with a veto scale of $20\,\mathrm{GeV}$, while the data collected during 2011 are used in a restricted rapidity range with a veto scale of $30\,\mathrm{GeV}$ but can access higher values of ${\overline{p_{\mathrm{T}}}}$.

The content of the paper is as follows. Section 2 describes the ATLAS detector followed by Sect. 3, which details the Monte Carlo simulation samples used. Jet reconstruction and event selection are presented in Sect. 4 and Sect. 5 respectively. The correction for detector effects is shown in Sect. 6 and discussion of systematic uncertainties on the measurement is in Sect. 7. Section 8 discusses the theoretical predictions before the results are presented in Sect. 9. Finally, the conclusions are given in Sect. 10.

## The ATLAS detector

ATLAS [44] is a general-purpose detector surrounding one of the interaction points of the LHC. The main detector components relevant to this analysis are the inner tracking detector and the calorimeters; in addition, the minimum bias trigger scintillators (MBTS) are used for selecting events during early data taking. The inner tracking detector covers the pseudorapidity range ${{|{\eta } |} <2.5}$ 1 and has full coverage in azimuthal angle. There are three main components to the inner tracker. In order, moving outwards from the beam-pipe, these are the silicon pixel detector, the silicon microstrip detector and the straw-tube transition–radiation tracker. These components are arranged in concentric layers and immersed in a $2\,\mathrm{T}$ magnetic field provided by the superconducting solenoid magnet.

The calorimeter is also divided into sub-detectors, providing overall coverage for ${{|{\eta } |} <4.9}$. The electromagnetic calorimeter, covering the region ${{|{\eta } |} <3.2}$, is a high-granularity sampling detector in which the active medium is liquid argon (LAr) interspaced with layers of lead absorber. The hadronic calorimeters are divided into three sections: a tile scintillator/steel calorimeter is used in both the barrel (${{|{\eta } |} <1.0}$) and extended barrel cylinders (${0.8<{|{\eta } |} <1.7}$) while the hadronic endcap (${1.5<{|{\eta } |} <3.2}$) consists of LAr/copper calorimeter modules. The forward calorimeter measures both electromagnetic and hadronic energy in the range ${3.2<{|{\eta } |} <4.9}$ using LAr/copper and LAr/tungsten modules.

The MBTS system consists of 32 scintillator counters, organized into two disks with one on each side of the detector. They are located in front of the end-cap calorimeter cryostats and cover the region ${2.1<{|{\eta } |} <3.8}$.

The online trigger selection used in this analysis employs the minimum bias and calorimeter jet triggers [45]. The minimum bias triggers are only available at the hardware level, while the calorimeter triggers have both hardware and software levels. The Level-1 (L1) hardware-based trigger provides a fast, but low-granularity, reconstruction of energy deposited in towers in the calorimeter; the Level-2 (L2) software implements a simple jet reconstruction algorithm in a window around the region triggered at L1; and finally the Event Filter (EF) performs a more detailed jet reconstruction procedure taking information from the entirety of the detector. The efficiency of jet triggers is determined using a bootstrap method, starting from the fully efficient MBTS trigger [45]. Between March and August of 2010, only L1 information was used to select events; both the L1 and L2 stages were used for the remainder of the 2010 data-taking period and all three levels were required for data taken during 2011.

## Monte Carlo event simulation

Simulated proton–proton collisions at ${\sqrt{s} = 7\,\mathrm{TeV}}$ were generated using the pythia 6.4 [46] program. These were used only to derive systematic uncertainties and to correct for detector effects; for this purpose they are compared against uncorrected data. Additional samples used to compare theoretical predictions to the data are described in Sect. 8.

The pythia program implements leading-order (LO) QCD matrix elements for ${2\rightarrow 2}$ processes followed by ${p_{\mathrm{T}}}$-ordered parton showers and the Lund string hadronisation model. The underlying event in pythia is modelled by multiple-parton interactions interleaved with the initial-state parton shower.

The events were generated using the MRST LO* parton distribution functions (PDFs) [47, 48]. Samples which simulated the data-taking conditions during 2010 (2011) used version 6.423 (6.425) of the generator, together with the ATLAS AMBT1 [49] (Perugia 2011 [50]) underlying event tune. For the samples simulating the data-taking conditions from 2011, additional $pp$ collisions were overlaid onto the hard scatter in the correct proportions to replicate this effect in the data. The final-state particles were passed through a detailed geant4  [51] simulation of the ATLAS detector [52] before being reconstructed using the same software used to process data.

## Jet reconstruction

The collision events selected by the ATLAS trigger system were fully reconstructed offline. Energy deposits in the calorimeter left by electromagnetic and hadronic showers were calibrated to the electromagnetic (EM) scale^2^. Three-dimensional topological clusters (“topoclusters”) [53] were constructed from seed calorimeter cells according to an iterative procedure designed to suppress electronic noise [54]. Each of these was then treated as a massless particle with direction given by its energy-weighted barycentre. The topoclusters were then passed as input to the FastJet  [55] implementation of the ${\mathrm{anti}\hbox {-}{k_t}}$ jet algorithm [56] with distance parameter ${R=0.6}$ and full four-momentum recombination.

The jets built by the ${\mathrm{anti}\hbox {-}{k_t}}$ algorithm were then calibrated in a multi-step procedure. Additional energy arising from “in-time pileup” (simultaneous $pp$ collisions within a single bunch crossing) was subtracted using a correction derived from data. Each event was required to have at least one primary vertex, reconstructed using two or more tracks, each with ${{p_{\mathrm{T}}} > 400\,\mathrm{MeV}}$ and the primary vertex with the highest ${\sum {p_{\mathrm{T}}^2}}$ of tracks associated with it was identified as the origin of the hard scatter. The jet position was recalibrated to point to this identified hard scatter primary vertex, rather than the geometric centre of the detector. A series of ${p_{\mathrm{T}}}$- and ${\eta }$-dependent energy correction factors derived from simulated events were used to correct for the response of the detector to jets. For the data collected during 2011, additional calibration steps were applied. Energy contributions, which were usually negative, coming from “out-of-time pileup” (residual electronic effects from previous $pp$ collisions) were corrected for using an offset correction derived using simulation.

A final in situ calibration, using ${Z}$+jet balance, ${\gamma }$+jet balance and multi-jet balance, was then applied to correct for residual differences in jet response between the simulation and data. The calibration procedure is described in more detail elsewhere [57, 58].

## Event selection

The measurements were performed using only the data from specific runs and run periods in which the detector, trigger and reconstructed physics objects satisfied data-quality selection criteria. Beam background was rejected by requiring at least one primary vertex in each event while selection requirements were applied to the hard-scatter vertex to minimise contamination from pileup. For data collected during 2010, the event was required to have only one primary vertex with five or more associated tracks; the proportion of such events was 93 % in the early low-luminosity runs, falling to 21 % in the high-luminosity runs at the end of the year. For data collected during 2011, the hard-scatter vertex was required to have at least three associated tracks. Jets arising from pileup were rejected using the jet vertex fraction (JVF). The JVF takes all tracks matched to the jet of interest and measures the ratio of ${\sum {p_{\mathrm{T}}}}$ from tracks which originated in the hard-scatter vertex to the ${\sum {p_{\mathrm{T}}}}$ of all tracks matched to the jet. For this analysis only jets with ${\text {JVF}>0.75}$ were used; all other jets were considered to have arisen from pileup and were therefore ignored. Due to the limited coverage of the tracking detectors, the JVF is only available for jets satisfying ${{|y|} < 2.4}$, which limits the acceptance in rapidity for 2011 data.

Due to the high instantaneous luminosity reached by the LHC, only high-threshold jet triggers remained unprescaled throughout the data-taking period in question. Jets with transverse momentum below the lowest-threshold unprescaled trigger were therefore only recorded using prescaled triggers. For data collected during 2011, ${\overline{p_{\mathrm{T}}}}$ was used to determine the most appropriate trigger to use for each event. Among all of the triggers determined to be fully efficient at the particular ${\overline{p_{\mathrm{T}}}}$ in question, the one which had been least prescaled was selected: only events passing this trigger were considered.

For the data collected during 2010, it was necessary to combine triggers from the central region (${{|{\eta } |} \le 3.2}$) and the forward region (${3.1 < {|{\eta } |} \le 4.9}$) of the detector, due to the large ${\Delta y}$ span under consideration. In each of these regions, efficiency curves as a function of jet transverse momentum were calculated for each trigger on a per-jet basis, rather than the per-event basis described above. The triggers were ordered according to their prescales and a lookup table was created, showing the point at which each trigger reached a plateau of 99 % efficiency.

An appropriate trigger was chosen for each of the two leading (highest transverse momentum) jets in each event; this was the lowest prescale trigger to have reached its efficiency plateau at the relevant ${p_{\mathrm{T}}}$ and ${|y|}$. The dijet event was then accepted if the event satisfied the trigger appropriate to the leading jet, the subleading jet or both. This procedure maximised event acceptance, since the random factor inherent in the trigger prescale meant that some events could be accepted based on the properties of the subleading jet even when the appropriate trigger for the leading jet had not fired. In order to combine overlapping triggers with different prescales, the procedure detailed in Ref. [59] was followed. In some less well-instrumented or malfunctioning regions of the detector, the per-jet trigger efficiency plateau occurred at less than the usual 99 % point. This introduced a measurable trigger inefficiency, which was corrected for by weighting events containing jets in these regions by the inverse of the efficiency.

Jets were required to have transverse momentum ${p_{\mathrm{T}}} > 20 (30)\,\mathrm{GeV}$ for data collected during 2010 (2011), thus ensuring that they remained in a region for which the jet energy scale had been evaluated (see Sect. 4). Jets were restricted in rapidity to ${{|y|} < 4.4}$ for data collected in 2010, with a stricter requirement of ${{|y|} < 2.4}$ applied in 2011 to ensure that the JVF could be determined for all jets. The two leading jets satisfying these criteria were then identified as the dijet system of interest. The event was rejected if the transverse momentum of the leading jet was below $60\,\mathrm{GeV}$ or if that of the subleading jet was below $50\,\mathrm{GeV}$. For the data collected during 2011, a minimum rapidity separation, ${{\Delta y} \ge 1}$, was required to enhance the physics of interest. The veto scale, ${Q_{0}}$, was set to ${{p_{\mathrm{T}}} > 20 (30)\,\mathrm{GeV}}$ for data collected in 2010 (2011).

Jet cleaning criteria [60, 61] were developed in order to reject fake jets, those which come from cosmic rays, beam halo or detector noise. These criteria also removed jets which were badly measured due to falling into poorly instrumented regions. Events collected in 2010 (2011) were rejected if they contained any jet with transverse momentum ${{p_{\mathrm{T}}} > 20 (30)\,\mathrm{GeV}}$ that failed these cleaning cuts. This requirement was also applied to the simulated samples where appropriate.

Additionally, a problem developed in the LAr calorimeter during 2011 running, resulting in a region in which energies were not properly recorded. As a result, a veto was applied to events that had at least one jet with ${{p_{\mathrm{T}}} > 30\,\mathrm{GeV}}$ falling in the vicinity of this region during the affected data-taking periods. This effect was replicated in the relevant simulation samples, which were reweighted to the data to ensure that an identical proportion of such events were included.

**Fig. 1:**
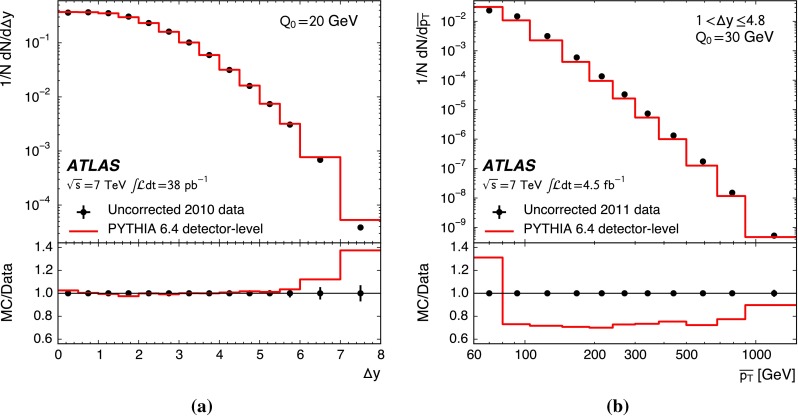
Comparison between uncorrected data (*black points*) and detector level pythia 6.4 Monte Carlo events (*solid line*). Statistical errors on the data are shown. The normalised distribution of events is presented as a function of **a** ${\Delta y}$ and **b** ${\overline{p_{\mathrm{T}}}}$. The ratio of the pythia 6.4 prediction to the data is shown in the *bottom panel*

In total, 1188583 events were accepted from the data collected in 2010, with 852030 of these being gap events: those with no additional jets above the veto scale in the rapidity interval between the dijet s. For data collected during 2011, 1411676 events were accepted, with 938086 of these being gap events. Data from 2010 and from 2011 were compared after being analysed separately and were found to agree to within the experimental uncertainties in a region kinematically accessible with both datasets. Figure 1 shows the comparison between detector level data and pythia 6.4 simulation in dijet events. The normalised number of events is presented as a function of ${\Delta y}$ in Fig. 1a and of ${\overline{p_{\mathrm{T}}}}$ in Fig. 1b. In both cases, and in all similar distributions, the pythia 6.4 event generator and geant4 detector simulation give a fair description of the uncorrected data.

## Correction for detector effects

Before comparing to theoretical predictions, the data are corrected for all experimental effects so that they correspond to the particle-level final state. This comprises all stable particles, defined as those with a proper lifetime longer than $10\,\mathrm{ps}$, including muons and neutrinos from decaying hadrons [62]. The correction for detector resolutions and inefficiencies is made by unfolding the measured distributions using the Bayesian procedure [63] implemented in the RooUnfold framework [64].

Bayesian unfolding entails using simulated events to calculate a transfer matrix which encodes bin-to-bin migrations between particle-level distributions and the equivalent reconstructed distributions at detector level. A series of bin transition probabilities are obtained from the matrix, and Bayes’ theorem is used to calculate the corresponding inverse probabilities; the process is then repeated iteratively. In this paper, the unfolding is performed using the pythia 6.4 samples described in Sect. 3 and the number of iterations is set to two throughout, as this was found to be sufficient to achieve convergence.

As the results shown here are constructed from multi-dimensional distributions, this must also be taken into account when unfolding. Each distribution is unfolded in two or three dimensions, with these dimensions being relevant combinations of ${\Delta \phi }$, ${\Delta y}$, ${\overline{p_{\mathrm{T}}}}$, ${\cos \left( \pi - \Delta \phi \right) }$, ${\cos \left( 2 \Delta \phi \right) }$ and the classification of the event as gap or non-gap. This means that the transfer matrices are four-dimensional or six-dimensional, rather than the usual two-dimensional case. This allows the effect of all possible bin migrations to be evaluated.

The statistical uncertainties are estimated by performing pseudo-experiments [65]. Each event in data is assigned a weight drawn from a Poisson distribution with unit mean for each pseudo-experiment and these weighted events are used to build a series of one thousand replicas for each distribution. Each of these replicas is unfolded, and the root-mean-squared spread around the nominal value is used to measure the statistical error on the unfolded result.

Possible bias arising from mismodelling of the distributions considered here is evaluated by performing a self-consistency check using pythia 6.4 events. The pythia 6.4 simulation is reweighted on an event-by-event basis using a three-dimensional function which is chosen in such a way as to ensure that the output of this reweighting step will approximate the uncorrected detector level data. For the pythia sample simulated to replicate 2010 data-taking conditions, the reweighting is carried out as a function of ${\Delta y}$, ${\Delta \phi }$ and the highest ${p_{\mathrm{T}}}$ among jets in the rapidity interval; for the sample simulated to replicate 2011 data-taking conditions, ${\overline{p_{\mathrm{T}}}}$ is used instead of ${\Delta y}$. This reweighted detector level pythia 6.4 sample is then unfolded using the original transfer matrix and the result is compared with the particle-level spectrum, which was itself implicitly modified through the event-by-event reweighting. Any remaining difference between these distributions is then taken as a systematic uncertainty associated with the unfolding procedure.

## Systematic uncertainties

For the most part, the dominant systematic uncertainty on these measurements is the one coming from the jet energy scale (JES) calibration. This uncertainty was determined using a combination of in situ calibration techniques, as detailed earlier, test-beam data and Monte Carlo modelling [57, 58]. It comprises 13 independent components for data taken in 2010 and 64 components for the 2011 data. The uncertainty components fully account for the differences in jet calibration discussed in Sect. 4, thus ensuring that data collected in the two years are fully compatible within their uncertainties.

For each component, all jet energies and transverse momenta are shifted up or down by one standard deviation of the uncertainty and the shifted jets are then passed through the full analysis chain. The measured distributions are unfolded and compared to the nominal distribution; the difference between these is taken as the uncertainty for the component in question. These fractional differences are then combined in quadrature, since the components are uncorrelated, to compute the jet energy scale uncertainty.

The uncertainty on the energy resolution of jets (JER) is derived in situ, using dijet balance techniques and the bisector method; it is then cross-checked through comparison with simulation [66]. Jet angular resolutions are estimated using simulated events and cross-checked using in situ techniques, where good agreement is observed with the simulation. These resolution uncertainties are propagated through the unfolding procedure by smearing the energy or angle of each reconstructed jet in each simulated event by a Gaussian function, with its width given by the quadratic difference between the nominal resolution and the resolution after shifting by the resolution uncertainty. This procedure is repeated one thousand times for each jet, to remove the effects of statistical fluctuations. The resulting smeared events are used to calculate a modified transfer matrix incorporating the resolution uncertainty; this matrix is then used to unfold the data. The ratio of this distribution to the distribution unfolded using the nominal transfer matrix is taken as a systematic uncertainty.

The trigger efficiency correction which is applied to events with jets falling into poorly measured detector regions also has an associated systematic uncertainty. This is determined by increasing or decreasing the measured inefficiencies by an absolute shift of 10 %, with a maximum efficiency of 100 %. The full correction procedure is then carried out using these new correction factors and the difference between this and the nominal distribution is taken as a systematic uncertainty on the measurement.

The effect of statistical fluctuations in the samples used to derive these uncertainties is also estimated by performing pseudo-experiments. Each event in the sample is assigned a weight drawn from a Poisson distribution with unit mean for each pseudo-experiment and these weighted events are used to build a series of one thousand replicas of the transfer matrices. These replicas are then used to unfold the nominal data sample; the root-mean-squared spread around the nominal value provides the systematic error due to the limited statistical precision of the Monte Carlo samples used.

For each of the systematic variations considered, the pseudo-experiment approach applied to the data is used to determine statistical uncertainties on each distribution and correlations between bins. Each fractional uncertainty was smoothed to remove these statistical fluctuations before the uncertainties were combined. To do this, each systematic component was rebinned until each bin showed a statistically significant deviation from the nominal value. This rebinned distribution was then smoothed using a Gaussian kernel and the smoothed function was evaluated at each of the original set of bin centres. The overall fractional uncertainty was then obtained by summing the individual sources in quadrature.

The uncertainty on the unfolding procedure, estimated as described in Sect. 6 is also important in some distributions. There is also an additional uncertainty on the luminosity calibration which is not included here. For the cross section s this is $3.5\,\%$ while it cancels for all other distributions.

Other sources of uncertainty were examined, found to be negligible and therefore ignored. Specifically, residual pileup contributions from soft-scatter vertices were studied by dividing the data into two subsamples coming from high-luminosity and low-luminosity runs. The disagreement between these subsamples was found to be negligible and hence no separate systematic uncertainty was assigned. The effect of varying the cut applied on the JVF was also studied and was found to produce differences in the detector-level distributions. However, such differences were well-reproduced by the pythia 6.4 sample and the resulting deviations after unfolding were much smaller than other uncertainties. Accordingly, no systematic uncertainty was assigned here either.

**Fig. 2:**
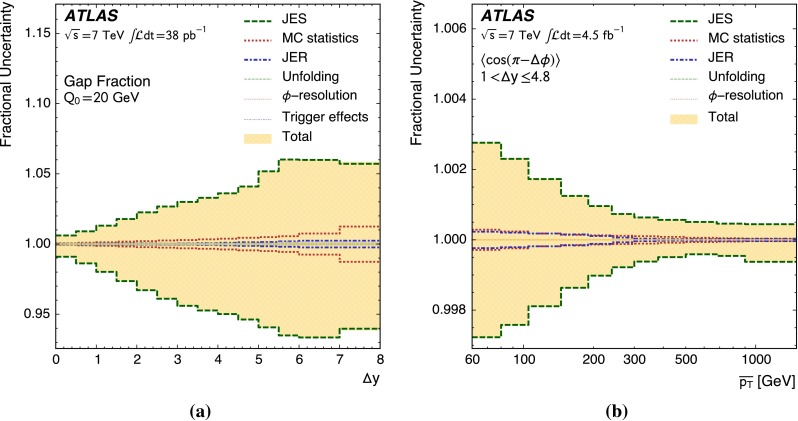
Summary of systematic uncertainties on **a** the gap fraction as a function of ${\Delta y}$ and **b** the ${\langle \cos \left( \pi - \Delta \phi \right) \rangle }$ distribution as a function of ${\overline{p_{\mathrm{T}}}}$. Here the systematic uncertainties from the jet energy scale (*dark dashes*), Monte Carlo statistical precision (*dark dots*), jet energy resolution (*dark dashed-dots*), unfolding (*light dashes*), jet ${\phi }$ resolution (*light dots*) and from residual trigger inefficiencies (*light dashed-dots*) are shown. The total systematic uncertainty (*light cross-hatched area*) is also shown

Figure 2 shows the summary of systematic uncertainties for two sample distributions: Fig. 2a for the gap fraction as a function of ${\Delta y}$ and Fig. 2b for the ${\langle \cos \left( \pi - \Delta \phi \right) \rangle }$ distribution as a function of ${\overline{p_{\mathrm{T}}}}$.

## Theoretical predictions

Two state-of-the-art theoretical predictions, namely High Energy Jets (HEJ) [16, 67] and the powheg  Box [68–70], are considered in this paper.

hej provides a leading-logarithmic (LL) calculation of the perturbative terms that dominate the production of multi-jet events when the jets span a large range in rapidity [16, 67, 71]. This formalism resums logarithms relevant in the Mueller–Navelet [72] limit, and incorporates a contribution from all final states with at least two hard jets. The purely partonic multi-jet output from hej can also be interfaced to the ariadne parton shower framework [73] to evolve the prediction to the hadron-level final state [74]. The ariadne program is based on the colour-dipole cascade model [75] in which gluon emissions are modelled as radiation from colour-connected partons, and provides soft and collinear radiation down to the hadronic scale, using pythia 6.4 for hadronisation. This accounts for radiation in the rapidity region outside the multi-jet system modelled by hej and represents a contribution from small-$x$, BFKL-like, logarithmic terms.

The powheg Box (version r2169) provides a full next-to-leading-order dijet calculation and is interfaced to either pythia 8  [76] (AU2 tune with $\alpha _\text {s}$ matching for the ISR [77]) or herwig  [78] (AUET1 tune [79]) to provide all-order resummation of soft and collinear emissions using the parton shower approximation. The advantage over the simple ${2\rightarrow 2}$ matrix elements is that the emission of an additional third hard parton is calculated exactly in pQCD, allowing observables that depend on the third jet to be calculated with good accuracy [80].

The prediction provided by powheg uses the DGLAP formalism, while that provided by hej is based on BFKL. For all theoretical predictions, events were generated using the CT10 PDF set [81]. The orthogonal error sets provided as part of the CT10 PDF set were used in order to evaluate the uncertainty inherent in the PDF, at the 68 % confidence level, following the CTEQ prescription [82]. The default choice for the renormalisation and factorisation scales was the transverse momentum of the leading parton in each event. The uncertainty due to higher-order corrections was estimated for the powheg prediction by increasing and decreasing the scale by a factor of two and taking the envelope of these variations. For the hej predictions, an envelope of nineteen scale variations was considered. These were constructed by varying each scale upwards and downwards by factors of 2 and $\sqrt{2}$, but excluding those cases where the ratio between the two scale factors was greater than two.

The scale uncertainty and PDF uncertainties were combined in quadrature to construct an overall uncertainty for each prediction. The PDF uncertainties are small across all of the phase-space regions considered in this paper and hence the predominant contribution to the uncertainty comes from the scale uncertainty. Theoretical uncertainties on the hej+ariadne prediction are not currently calculable and are not shown here. The range covered by the central values of the powheg+pythia 8 and powheg+herwig predictions gives an estimate of the uncertainty inherent in the parton shower matching procedure. This range, together with the uncertainty band on the powheg+pythia 8 prediction, can be considered together as a total theoretical uncertainty on the NLO+DGLAP prediction that can be compared to the predictions given by hej and hej+ariadne.

Finally, in order to allow comparisons against the fully corrected data distributions presented in this paper, the partons from hej or the final-state particles from hej+ariadne, powheg+pythia 8 and powheg+herwig were clustered together using the same jet algorithm and parameters as for the data.

## Results and discussion

The fully corrected data are compared to next-to-leading-order theoretical predictions from powheg and hej, as explained in Sect. 8. The powheg prediction is presented after parton showering, hadronisation and underlying event simulation with either pythia 8 or herwig. As the uncertainties on these two powheg predictions are highly correlated, uncertainties are only shown on the powheg+pythia 8 prediction, with only the central value of the powheg+herwig prediction presented. Two hej curves are presented: one a pure parton-level prediction and the second after interfacing with the ariadne parton shower. The central value of the hej+ariadne prediction is shown, together with the statistical uncertainty on this prediction.

### Gap fraction and mean jet multiplicity

**Fig. 3:**
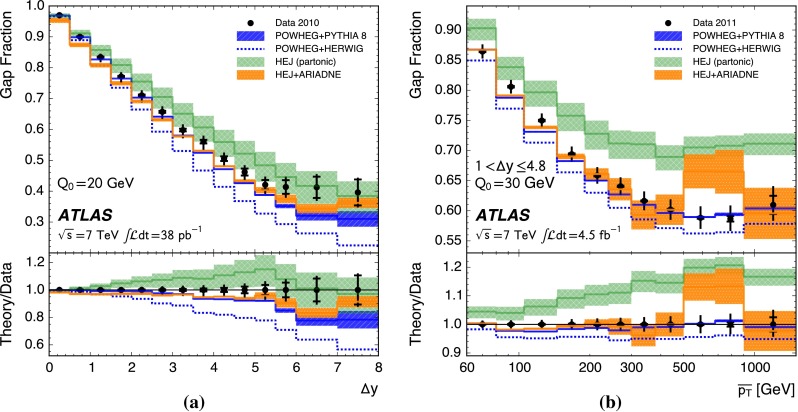
The measured gap fraction (*black dots*) as a function of **a** ${\Delta y}$ and **b** ${\overline{p_{\mathrm{T}}}}$. The *inner error bars* represent statistical uncertainty while the *outer error bars* represent the quadrature sum of the systematic and statistical uncertainties. For comparison, the predictions from parton-level hej (*light-shaded cross-hatched band*), hej+ariadne (*mid-shaded dotted band*), powheg+pythia 8 (*dark-shaded hatched band*) and powheg+herwig (*dotted line*) are also included. The ratio of the theory predictions to the data is shown in the *bottom panel*

**Fig. 4:**
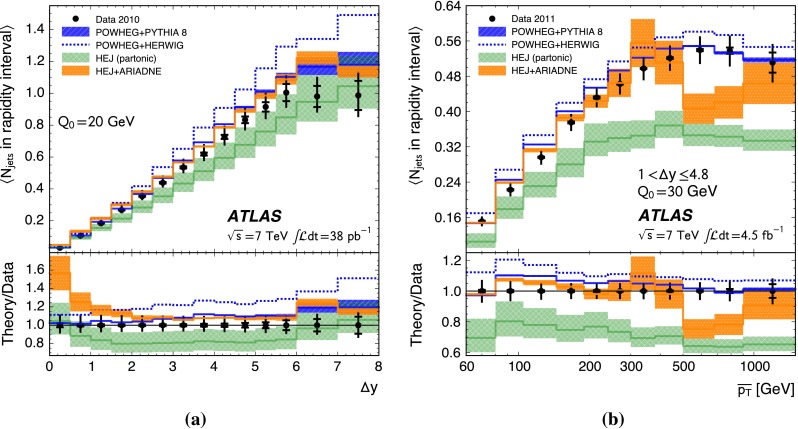
The mean number of jets above the veto threshold in the rapidity interval bounded by the dijet system measured in data as a function of **a** ${\Delta y}$ and **b** ${\overline{p_{\mathrm{T}}}}$. For comparison, the hej, hej+ariadne, powheg+pythia 8 and powheg+herwig predictions are presented in the same way as Fig. 3

Figures 3 and 4 show the gap fraction and the number of jets in the rapidity gap, respectively, as functions of ${\Delta y}$ and ${\overline{p_{\mathrm{T}}}}$. Naïvely, it is expected from pQCD that the number of events passing the jet veto should be exponentially suppressed as a function of ${\Delta y}$ and ${\ln {\left( \overline{p_{\mathrm{T}}}/Q_{0}\right) }}$ due to the exchange of colour in the ${t}$-channel [19]. However, non-exponential behaviour may become apparent in the tails of these distributions as the steeply falling parton distribution functions can reduce the probability of additional quark and gluon radiation from the dijet system and increase the gap fraction [15]. This can be understood by considering the behaviour at extreme values of ${\Delta y}$ or ${\overline{p_{\mathrm{T}}}}$, when all of the collision energy is used to create the dijet pair and little is available for additional radiation. As the gap fraction is expected to be smooth it must therefore begin increasing at some point, so as to reach unity when this kinematic limit is obtained. Furthermore, since the cross section for QCD colour-singlet exchange increases with jet separation [21], any contribution from such processes would also lead to an increase in the gap fraction at large ${\Delta y}$.

The data do indeed show exponential behaviour in Fig. 3 at low values of ${\Delta y}$ and ${\overline{p_{\mathrm{T}}}}$, but deviate from purely exponential behaviour at the highest values of ${\Delta y}$ and ${\overline{p_{\mathrm{T}}}}$, with the gap fraction reaching a plateau in both distributions. For the ${\overline{p_{\mathrm{T}}}}$ distribution, this plateau is qualitatively reproduced by all the predictions considered here, even those which do not provide good overall agreement with the data. The plateau observed in data for the ${\Delta y}$ distribution is not, however, as prominent in any of the theoretical predictions, which all continue to fall as ${\Delta y}$ increases. A similar excess was observed in previous experiments [28–35] and was attributed to colour-singlet exchange effects. However, here the spread of theoretical predictions is too large to allow definite conclusions to be drawn and improved calculations are needed before a quantitative statement can be made.

In the high-${\Delta y}$ region, both powheg predictions slightly underestimate the gap fraction and hence overestimate the mean jet multiplicity in the rapidity interval. Partonic hej slightly overestimates the gap fraction for intermediate values of ${\Delta y}$. Interfacing hej to ariadne improves the description of the data across the ${\Delta y}$ spectrum.

powheg+pythia 8, which resums soft and collinear emissions through the parton shower approximation, provides a good description of the gap fraction and the mean jet multiplicity distributions as a function of ${\overline{p_{\mathrm{T}}}}$. On the other hand, the powheg+herwig model, which also provides a similar resummation, consistently predicts too much jet activity across the ${\overline{p_{\mathrm{T}}}}$ range. Conversely, hej, which does not attempt to resum these soft and collinear terms, provides a poor description of the data in the large ${\ln {\left( \overline{p_{\mathrm{T}}}/Q_{0}\right) }}$ limit. Significantly improved agreement with the data is seen when interfacing hej to the ariadne parton shower model, which performs a resummation of these terms. In fact, the prediction from hej+ariadne is similar to that from powheg+pythia 8 for most values of ${\Delta y}$ and ${\overline{p_{\mathrm{T}}}}$.

### Azimuthal decorrelations

**Fig. 5:**
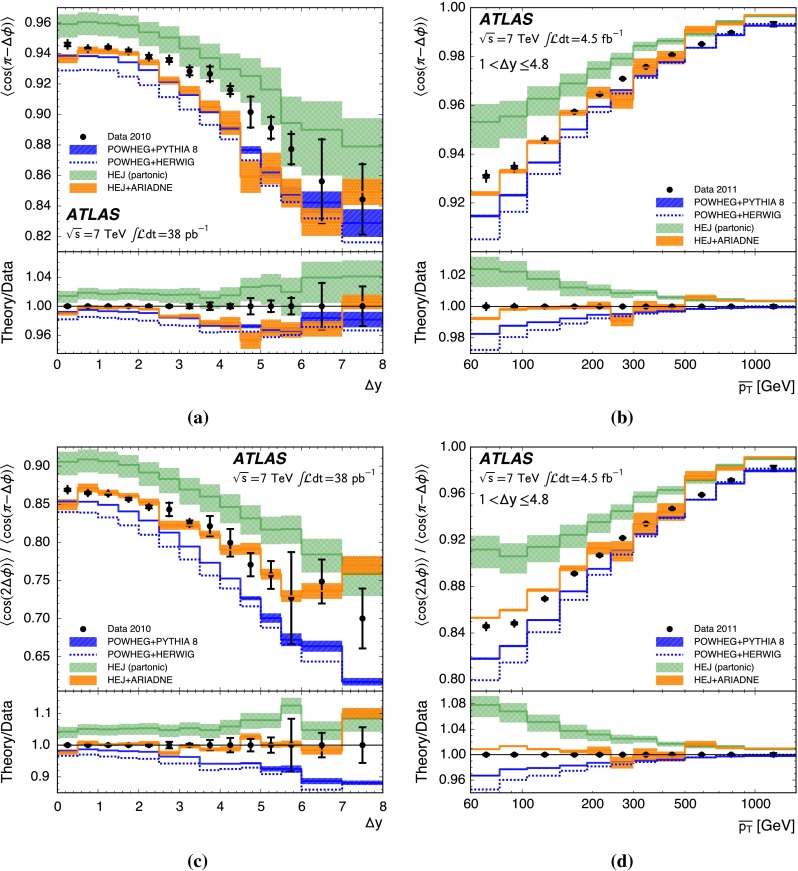
The measured **a**, **b** ${\langle \cos \left( \pi - \Delta \phi \right) \rangle }$ and **c**, **d** ${{\langle \cos \left( 2 \Delta \phi \right) \rangle }/ {\langle \cos \left( \pi - \Delta \phi \right) \rangle } \rangle }$ distributions as a function of **a**, **c** ${\Delta y}$ and **b**, **d** ${\overline{p_{\mathrm{T}}}}$. For comparison, the hej, hej+ariadne, powheg+pythia 8 and powheg+herwig predictions are presented in the same way as Fig. 3

Figure 5 shows the ${\langle \cos \left( \pi - \Delta \phi \right) \rangle }$ and ${\langle \cos \left( 2 \Delta \phi \right) \rangle }/ {\langle \cos \left( \pi - \Delta \phi \right) \rangle } \rangle$ distributions, as functions of ${\Delta y}$ and ${\overline{p_{\mathrm{T}}}}$, for inclusive dijet events. For the azimuthal moments, ${\langle \cos \left( n \left( \pi - \Delta \phi \right) \right) \rangle }$, a decrease (increase) in azimuthal correlation manifests as a decrease (increase) in the azimuthal moment. As the dijet s deviate from a back-to-back topology, the second azimuthal moment falls more rapidly than the first (in the region ${{\Delta \phi } > \pi /2}$ where the majority of events lie). The ratio ${{\langle \cos \left( 2 \Delta \phi \right) \rangle }/ {\langle \cos \left( \pi - \Delta \phi \right) \rangle } \rangle }$ is, therefore, expected to show a similar, but more pronounced, dependence on azimuthal correlation to that seen in the moments.

The data show the expected qualitative behaviour of a decrease in azimuthal correlation with increasing ${\Delta y}$ and increase in azimuthal correlation with increasing ${\overline{p_{\mathrm{T}}}}$. Both powheg predictions underestimate the degree of azimuthal correlation except in the high-${\overline{p_{\mathrm{T}}}}$ region, while hej predicts too much azimuthal correlation. In both cases, the changing degree of correlation with ${\Delta y}$ and ${\overline{p_{\mathrm{T}}}}$ is, for the most part, well described by the predictions. The largest differences between the predictions and the data are seen at high ${\Delta y}$ and low ${\overline{p_{\mathrm{T}}}}$.

Additionally, it can be seen that the separation between theoretical predictions for the ratio ${\langle \cos \left( 2 \Delta \phi \right) \rangle }/ {\langle \cos \left( \pi - \Delta \phi \right) \rangle } \rangle$ is significantly greater than for the ${\langle \cos \left( \pi - \Delta \phi \right) \rangle }$ distribution alone, considering the uncertainties of these predictions. This means that the ratio gives enhanced discrimination between the DGLAP-like powheg and BFKL-like hej predictions, as predicted by theoretical calculations [83]. Here, neither hej nor powheg provide good agreement with the data. However, the hej+ariadne prediction gives a good description of the data for both low-${\overline{p_{\mathrm{T}}}}$ and for ${\Delta y}$.

**Fig. 6:**
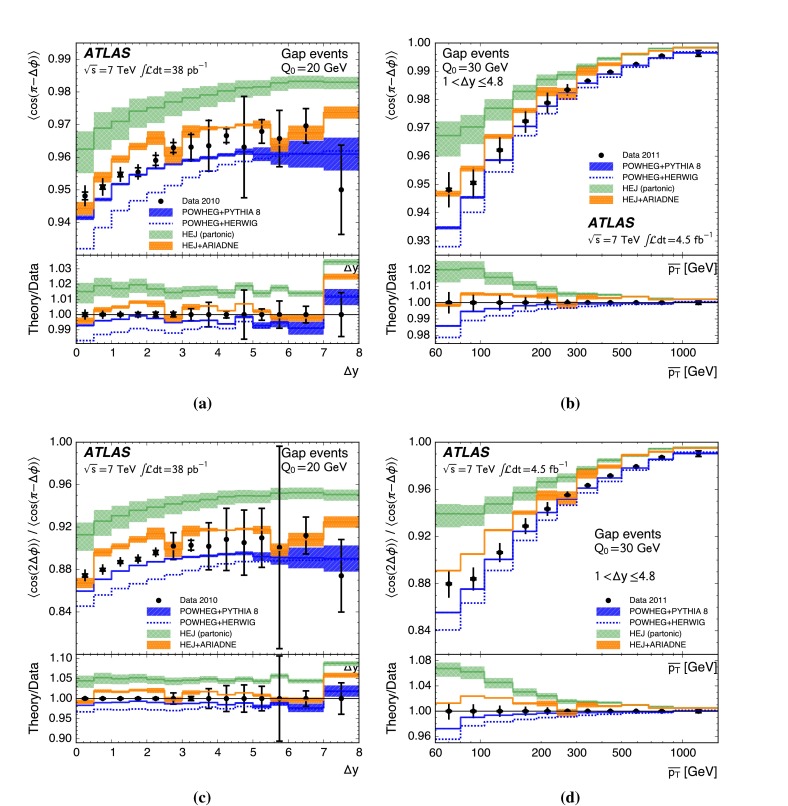
The measured **a**, **b** ${\langle \cos \left( \pi - \Delta \phi \right) \rangle }$ and **c**, **d** ${\langle \cos \left( 2 \Delta \phi \right) \rangle }/ {\langle \cos \left( \pi - \Delta \phi \right) \rangle } \rangle$ distributions, for gap events as a function of **a**, **c** ${\langle \cos \left( \pi - \Delta \phi \right) \rangle }$ ${\Delta y}$ and **b**, **d** ${\langle \cos \left( \pi - \Delta \phi \right) \rangle }$ ${\overline{p_{\mathrm{T}}}}$. The veto scale is ${Q_{0}}$ = 20 $(30)\,\mathrm{GeV}$ for data collected during 2010 (2011). For comparison, the hej, hej+ariadne, powheg+pythia 8 and powheg+herwig predictions are presented in the same way as Fig. 3

Figure 6 shows the corresponding ${\langle \cos \left( \pi - \Delta \phi \right) \rangle }$ and ${{\langle \cos \left( 2 \Delta \phi \right) \rangle }/ {\langle \cos \left( \pi - \Delta \phi \right) \rangle } \rangle }$ distributions for events that pass the veto requirement on additional jet activity in the rapidity interval bounded by the dijet system. In this case, with the jet veto suppressing additional quark and gluon radiation, the spectra show the opposite behaviour, namely a slight increase in correlation with ${\Delta y}$, which now agrees with the rise seen in the ${\overline{p_{\mathrm{T}}}}$ distribution. This can be explained by considering that as ${\Delta y}$ or ${\overline{p_{\mathrm{T}}}}$ increase, the veto requirement imposes an increasingly back-to-back topology on the dijet system. The spread of theoretical predictions is again large in each distribution, with the powheg predictions having too much decorrelation and hej predicting too little decorrelation.

The use of the ariadne parton shower again brings hej into better agreement with the data, although not as well as in the inclusive case. The best agreement is given by powheg+pythia 8, especially in the highest ${\overline{p_{\mathrm{T}}}}$ bins. hej agrees less well with the data than in the inclusive case, showing that this region of widely separated hard jets without additional radiation in the event is not well reproduced by the hej calculation. A quantitative statement about the degree of agreement seen here between hej+ariadne and the data cannot, however, be made in the absence of theoretical uncertainties on this calculation.

**Fig. 7:**
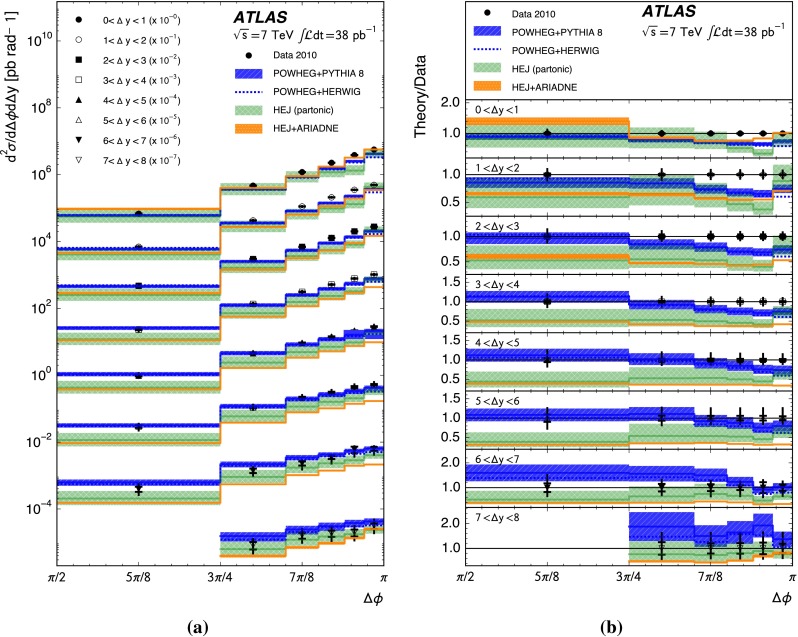
The measured double-differential cross section s (*black points*) as a function of ${\Delta \phi }$ for eight slices in ${\Delta y}$. For comparison, the hej, hej+ariadne, powheg+pythia 8 and powheg+herwig predictions are presented in the same way as Fig. 3. In **a** the absolute comparison is shown, while in **b** the ratios of the predictions to the data are shown

**Fig. 8:**
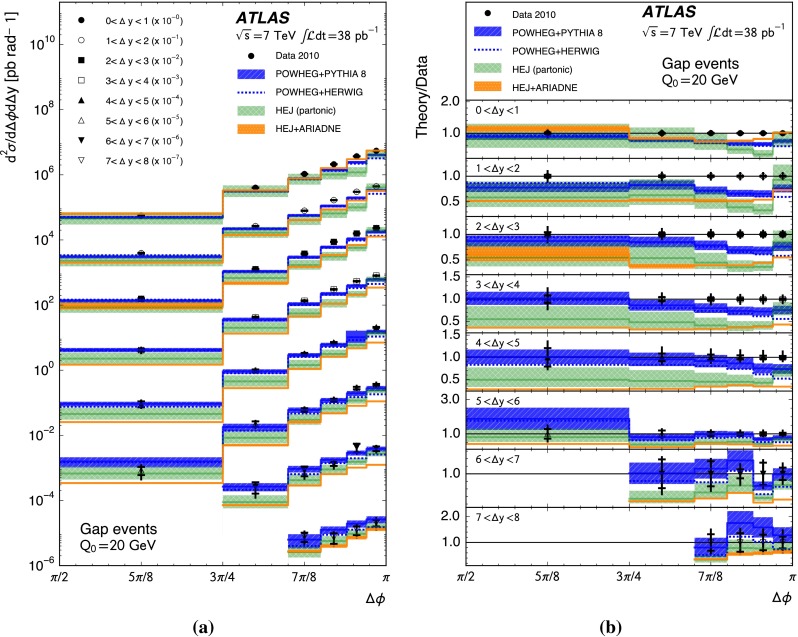
The measured double-differential cross section s as a function of ${\Delta \phi }$ for eight slices in ${\Delta y}$, for gap events. The veto scale is ${{Q_{0}} = 20\,\mathrm{GeV}}$. For comparison, the hej, hej+ariadne, powheg+pythia 8 and powheg+herwig predictions are presented in the same way as Fig. 3. In **a** the absolute comparison is shown, while in **b** the ratios of the predictions to the data are shown

Figures 7 and 8 show the double-differential dijet cross section s as functions of ${\Delta \phi }$ and ${\Delta y}$ for inclusive and gap events respectively. The predictions from powheg+pythia 8 and powheg+herwig provide a good overall description of the measured cross section s, within the experimental and theoretical uncertainties, with the only notable deviations occurring at high ${\Delta \phi }$ in the lowest ${\Delta y}$ bins. This is in agreement with the observations in previous ATLAS studies [1]. hej underestimates the cross section seen in data throughout the ${\Delta y}$ range, although it provides a good description of the overall shape. This underestimate is noticeably enhanced when only gap events are considered, which is a regime far from the wide-angle, hard-emission limit for which the underlying resummation procedure is valid.

## Summary

Theoretical predictions based on perturbative QCD are tested by studying dijet events in extreme regions of phase space. Measurements of the gap fraction, as a function of both the rapidity separation and the average dijet transverse momentum, together with the azimuthal decorrelation are presented as functions of ${\Delta y}$ and ${\overline{p_{\mathrm{T}}}}$, extending previous studies up to eight rapidity units in ${\Delta y}$ and $1.5\,\mathrm{TeV}$ in ${\overline{p_{\mathrm{T}}}}$. The measurements are used to investigate the predicted breakdown of DGLAP evolution and the appearance of BFKL effects by comparing the data to the all-order resummed leading-logarithmic calculations of hej and the full next-to-leading-order calculations of powheg. The full data sample collected with the ATLAS detector in $7\,\mathrm{TeV}$ $pp$ collisions at the LHC is used, corresponding to integrated luminosities of ${36.1}\,\mathrm{pb}^{-1}$ and ${4.5}\,\mathrm{fb}^{-1}$ for data collected during 2010 and 2011 respectively.

The data show the expected behaviour of a reduction of gap events, or equivalently, an increase in jet activity, for large values of ${\overline{p_{\mathrm{T}}}}$ and ${\Delta y}$, together with an associated rise in the number of jets in the rapidity interval. The azimuthal moments show an increase in correlation with increasing ${\overline{p_{\mathrm{T}}}}$ and an increase in (de)correlation with increasing ${\Delta y}$ for gap (all) events. The expected increase in cross section with ${\Delta \phi }$ is also seen.

The powheg+pythia 8 prediction provides a reasonable description of the data in most distributions, but shows disagreement in some areas of phase-space, particularly for the inclusive azimuthal distributions in the limit of large ${\Delta y}$ or small ${\overline{p_{\mathrm{T}}}/Q_{0}}$. When the herwig parton shower is used instead of pythia 8, the agreement with data worsens as herwig predicts too many jets above the veto scale. The partonic hej prediction provides a poor description of the data in most of the distributions presented, with the exception of the gap fraction and jet multiplicity distributions as a function of ${\Delta y}$. The addition of the ariadne parton shower, which accounts for some of the soft and collinear terms ignored in the hej approximation, brings the prediction closer to powheg+pythia 8.

No single theoretical prediction is able to simultaneously describe the data over the full phase-space region considered here; in general, however, the best agreement is given by powheg+pythia 8 and hej+ariadne. The variable best able to discrimate between the DGLAP-like prediction from powheg+pythia 8 and the BFKL-like prediction from hej is ${{\langle \cos \left( 2 \Delta \phi \right) \rangle }/ {\langle \cos \left( \pi - \Delta \phi \right) \rangle } \rangle }$. Here, it can clearly be seen that neither of these predictions describe the data accurately as a function of either ${\Delta y}$ or ${\overline{p_{\mathrm{T}}}}$. It should be noted, however, that when the inclusive event sample is considered, the hej+ariadne model, a combination of BFKL-like parton dynamics with the colour-dipole cascade model, provides a good description of ${{\langle \cos \left( 2 \Delta \phi \right) \rangle }/ {\langle \cos \left( \pi - \Delta \phi \right) \rangle } \rangle }$ in the large ${\Delta y}$ and small ${\overline{p_{\mathrm{T}}}}$ regions, where the powheg models show some divergence from the data.

In most of the phase-space regions presented, the experimental uncertainty is smaller than the spread of theoretical predictions. These disparities between predictions represent a genuine difference in the modelling of the underlying physics and the data can, therefore, provide a crucial input for constraining parton-shower models in the future—particularly in the case of QCD radiation between widely separated or high transverse momentum dijet s. Improved theoretical predictions are essential before any conclusions can be drawn about the presence or otherwise of BFKL effects or colour-singlet exchange in these data.

## Footnotes

1 ATLAS uses a right-handed coordinate system with its origin at the nominal interaction point (IP) in the centre of the detector and the ${z}$-axis along the beam pipe. The ${x}$-axis points from the IP to the centre of the LHC ring, and the ${y}$-axis points upward. Cylindrical coordinates ${(r,\phi )}$ are used in the transverse plane, ${\phi }$ being the azimuthal angle around the beam pipe. The pseudorapidity is defined in terms of the polar angle ${\theta }$ as ${\eta =-\ln \tan (\theta /2)}$. The rapidity of a particle with respect to the beam axis is defined as ${y = (1/2)\ln \left[ (E+p_z)/(E-p_z)\right] }$.

2 The electromagnetic scale is the basic calorimeter signal scale for the ATLAS calorimeters. It gives the correct response for the energy deposited in electromagnetic showers, but it does not correct for the different response for hadrons.
